# Hierarchical Bayesian estimation of covariate effects on airway and alveolar nitric oxide

**DOI:** 10.1038/s41598-021-96176-z

**Published:** 2021-08-25

**Authors:** Jingying Weng, Noa Molshatzki, Paul Marjoram, W. James Gauderman, Frank D. Gilliland, Sandrah P. Eckel

**Affiliations:** Department of Population and Public Health Sciences, University of Southern California, 2001 N. Soto Street, SSB 202B, MC-9234, Los Angeles, CA 90089 USA

**Keywords:** Statistics, Asthma, Biomarkers, Epidemiology

## Abstract

Exhaled breath biomarkers are an important emerging field. The fractional concentration of exhaled nitric oxide (FeNO) is a marker of airway inflammation with clinical and epidemiological applications (e.g., air pollution health effects studies). Systems of differential equations describe FeNO—measured non-invasively at the mouth—as a function of exhalation flow rate and parameters representing airway and alveolar sources of NO in the airway. Traditionally, NO parameters have been estimated separately for each study participant (Stage I) and then related to covariates (Stage II). Statistical properties of these two-step approaches have not been investigated. In simulation studies, we evaluated finite sample properties of existing two-step methods as well as a novel Unified Hierarchical Bayesian (U-HB) model. The U-HB is a one-step estimation method developed with the goal of properly propagating uncertainty as well as increasing power and reducing type I error for estimating associations of covariates with NO parameters. We demonstrated the U-HB method in an analysis of data from the southern California Children’s Health Study relating traffic-related air pollution exposure to airway and alveolar airway inflammation.

## Introduction

Exhaled breath biomarkers are an important emerging field, since they can be measured non-invasively and repeatedly^1^. The fractional concentration of exhaled nitric oxide (FeNO) is a marker of airway inflammation with applications in clinical settings (e.g., asthma^2^) and epidemiological research (e.g., studies of inhaled environmental exposures^3,4^). The nature of the non-invasive assessment of FeNO results in challenges that can be at least partially addressed by innovations in statistical methodology.

FeNO concentrations are inversely related to expiratory flow rate, suggesting both an airway and an alveolar source of nitric oxide (NO)^5,6^. High flow FeNO primarily reflects low NO concentrations in the alveolar region while low flow FeNO primarily reflects higher NO concentrations in the airway tissue^7,8^. Several mathematical models have been developed to describe the dynamics of NO in the lower respiratory tract, using systems of differential equations with parameters describing NO sources in the airway and alveolar compartments^9^. Conventional standardized assessment of FeNO at a fixed 50 ml/s flow rate^2,10,11^ treats flow dependency as a nuisance. However, measurement of FeNO at multiple expiratory flow rates (“multiple flow FeNO”) is a promising technique that takes advantage of the information across flow rates to non-invasively assess airway and alveolar inflammation using estimated parameters quantifying airway and alveolar source of NO. The growing interest in multiple flow FeNO has resulted in its inclusion in the most recent update to the guidelines for FeNO assessment^12^.

Non-invasive assessment of airway and alveolar NO has also sparked interest in relating estimated airway and/or alveolar NO to covariates, such as respiratory diseases^13,14^ and environmental exposures, including: ambient air pollution^15,16^, traffic-related air pollution^17^ and pollen^18^. All of these studies have used a “Two-Stage” (TS) approach to estimating the associations of covariates with NO parameters. Stage I consists of estimating NO parameters for each participant (using a variety of methods, which we will describe later). Stage II consists of relating each estimated NO parameter (treated as a known outcome) to covariates in a standard linear regression model. However, the statistical properties of the TS approach have not been thoroughly investigated.

The TS approach ignores the uncertainty in the Stage I estimated NO parameters when they are used in Stage II, thereby failing to propagate the statistical uncertainty. Additionally, TS methods may fail to produce NO parameter estimates in Stage I for some participants due to small sample size (e.g., in our motivating example there are ~ 9 observations/participant)^19^. Subsequent exclusion of these subjects in Stage II may bias the estimated association in Stage II. Another issue is that the Stage I model (i.e., the model estimating NO parameters) is only an approximation of reality and may include various assumptions aimed at simplification which impact parameter estimation (e.g., alveolar NO estimation has been shown to be sensitive to applying a first, second, or third order approximation to an exponential function in the Stage I model)^19^. To overcome the weakness of TS approaches, we had previously explored a nonlinear mixed effect (NLME) unified model to simultaneously estimate NO parameters and their associations with covariates. However, the method suffered from convergence issues^20^. For NLME models, which are widely-used in the field of pharmacokinetics, convergence issues are not uncommon and can sometimes be overcome through the careful choice of starting values^19,21^.

This paper presents a novel Unified Hierarchical Bayesian (U-HB) model for NO parameter estimation, based on previous statistical methodology developments in pharmacokinetics^21^ and implemented using Gibbs sampling^22^. Our U-HB model simultaneously estimates NO parameters and their associations with covariates, thereby propagating uncertainty throughout the entire analysis. The U-HB model is a novel application of Hierarchical Bayesian methods to the field of multiple flow FeNO. We present simulation studies evaluating the statistical properties of existing two-step methods and the novel U-HB approach. We then show results from an application of these methods to data from the southern California Children’s Health Study (CHS). The CHS is a landmark cohort study on the effects of air pollution exposures on children’s respiratory health^23–25^. The methodological work in this paper is motivated by the need for better statistical methods to address CHS research questions. In particular, our group has been interested in evaluating the effects of traffic-related air pollution exposures on airway and alveolar inflammation. Traffic is a major source of anthropogenic air pollution which is increasingly recognized as impacting human health^26–32^.

## Methods

We begin by introducing the deterministic steady-state two-compartment model for FeNO and then discuss a variety of TS approaches which have been used to estimate both the alveolar and airway NO parameters from this model and associations with covariates. We then introduce our novel U-HB approach.

### Deterministic, steady-state two-compartment model for FeNO

Our modeling work is based on the simple steady-state two-compartment model (henceforth referred to as the 2CM) which assumes a cylindrically-shaped airway compartment and an expansile alveolar compartment^9,33^. Under the 2CM, FeNO can be related to parameters quantifying airway and alveolar NO sources as follows:

1 $${\text{FeNO}} = C_{aw} + \left( {C_{A} - C_{aw} } \right) \times e^{{ - D_{aw} /flow}}$$

Equation 1 describes how FeNO (ppb) measured at the mouth is related to expiratory flow rate, “flow” (mL/s), and three “NO parameters”: *C*_*A*_, the concentration of NO in the alveolar region (ppb); *C*_*aw*_, the concentration of NO in the airway tissue (ppb); and *D*_*aw*_, the airway tissue diffusion capacity (pL·s^−1^·ppb^−1^). Note that another widely used parameter *J’*_*aw*_ , the maximum flux of NO in the airway, is the product of *C*_*aw*_ and *D*_*aw*_^9^. A number of common methods for estimating NO parameters in the 2CM use an alternative *J’*_*aw*_ parameterization of Eq. (1), but here we use the *C*_*aw*_ parameterization since it allows for direct estimation of a more interpretable parameter.

### Estimating NO parameters from the 2CM

The model in Eq. (1) is deterministic and nonlinear. To estimate 2CM parameters from stochastically observed multiple flow FeNO data (steady state summaries of repeated FeNO maneuvers at a range of target flow rates), researchers have relied on linear regression methods under various linearizing assumptions or nonlinear regression methods. For example, previous studies have used approximations based on Taylor expansions to the exponential function, including first order linear approximation methods (linT and linP)^9^, second order quadratic approximation methods (quadT and quadP)^19^, and a third order approximation method, the Högman and Merilӓinen algorithm (HMA)^13,34^. Nonlinear approaches include standard nonlinear least squares regression^35^ which essentially adds a random normal error to Eq. (1), as well as a natural log transform-both-sides extension^19^. The log transform-both-sides model acknowledges the increased variation in residuals that occurs as flow rate (and hence FeNO concentration) increases.

### Estimating associations of covariates with NO parameters

#### TS methods

We selected three existing TS approaches to be evaluated in a simulation study, along with the new U-HB method. In each of the following TS methods, Stage I estimates of the three NO parameters for participant *i* (${\widehat{{C}_{A}}}_{i}$, ${\widehat{{logC}_{aw}}}_{i}$, and ${\widehat{{logD}_{aw}}}_{i}$) are treated as known values and used as the outcomes in three separate Stage II linear regression models relating each NO parameter to a covariate X:

2 $$\begin{array}{*{20}l} {\widehat{{C_{A_i} }} = \alpha_{{C_{A} }} + \beta_{{C_{A} }} X_{i} + \varepsilon_{{C_{A_i} }} } \\ {\widehat{{\log C_{aw_i} }} = \alpha_{{\log C_{aw} }} + \beta_{{\log C_{aw} }} X_{i} + \varepsilon_{{\log C_{aw_i} }} } \\ {\widehat{{\log D_{aw_i} }} = \alpha_{{\log D_{aw} }} + \beta_{{\log D_{aw} }} X_{i} + \varepsilon_{{\log D_{aw_i} }} } \\ \end{array}$$

Henceforth, we will refer to the three TS approaches by the names of their Stage I models, which are:

1. The TS Högman and Merilӓinen Algorithm (TS-HMA). HMA^13,34^ is a widely-applied method for estimating NO parameters using an iterative algorithm involving a third-order approximation to the 2CM. In a study with N participants, Stage I consists of fitting N HMA models, one per participant. Stage I input data for each participant consists of 3 observations: average FeNO measured at low, medium, and high flow rates. In the CHS we use 30, 100, and 300 mL/s respectively^19^.
2. The TS Nonlinear Least Squares model (TS-NLS). The natural log transform-both-sides nonlinear least squares model proposed previously by our group^19^, and henceforth referred to as NLS, is implemented using standard nonlinear-least squares software (“nls” from the nlme package in R). The log transform-both-sides approach better satisfies the assumption of normally distributed errors while maintaining the physiological interpretation of the NO parameters^19^. For N participants, Stage I consists of fitting N log transform-both-sides NLS models of the following form, based on Eq. (1):
  3 $$\log \left( {FeNO} \right) = \log \left( {\exp \left( {\log C_{aw} } \right) + \left( {C_{A} - \exp \left( {\log C_{aw} } \right)} \right) \times e^{{ - \exp \left( {\log D_{aw} } \right)/flow_{j} }} } \right) + \varepsilon_{j}$$
  where *j* = 1*,…n*_*i*_ indexes the observations for each participant. In the CHS, the protocol asked each participant to perform 9 maneuvers, but in practice participants had between 4 and 12 valid maneuvers each. Each maneuver is summarized by a paired observation of FeNO concentration and flow^36^. Note that in Eq. (3), two NO parameters are expressed (and directly estimated) as ${{\text{log}}C}_{aw}$ and ${{\text{log}}D}_{aw}$to enforce positivity of ${C}_{aw}$ and ${D}_{aw}$ and to better satisfy the normality assumptions in the TS-NLME method (below). This is common practice in pharmacokinetics modeling^37^.
3. The TS Nonlinear Mixed Effect model (TS-NLME): In a nonlinear mixed effects (NLME) approach, we use FeNO maneuvers from all participants to estimate a single NLME model of the form:
  4 $$\log \left( {{\text{FeNO}}_{{ij}} } \right) = \log (\exp \left( {\log C_{{aw_i}} } \right) + \left( {C_{{A_{i} }} - \exp \left( {\log C_{{aw_i}} } \right)} \right) \times e^{{ - \exp \left( {\log D_{{aw_i}} } \right)/flow_{{ij}} }} ) + \varepsilon _{{ij}}$$
  with participant-level random effects which follow a multivariate normal distribution. Participant-level estimates of NO parameters are obtained by combining fixed effect parameter estimates with empirical Bayes estimates of the random effects. Because TS-NLME fits a single model (rather than N models) in Stage I, it does not suffer from the small sample size issues that affect TS-HMA or TS-NLS (each of which often fails to produce estimates for a subset of the population at Stage I). In TS-NLME, which has been applied previously^17^, NO parameters estimated in a Stage I NLME are then related to covariates in a separate Stage II.

In secondary analyses, we evaluated two alternative TS-NLS approaches: a constrained version of TS-NLS (in which we assume ${C}_{A}$ > 0.001) and a version of TS-NLS with an inverse-variance weighted Stage II.

#### Unified methods

**Figure 1:**
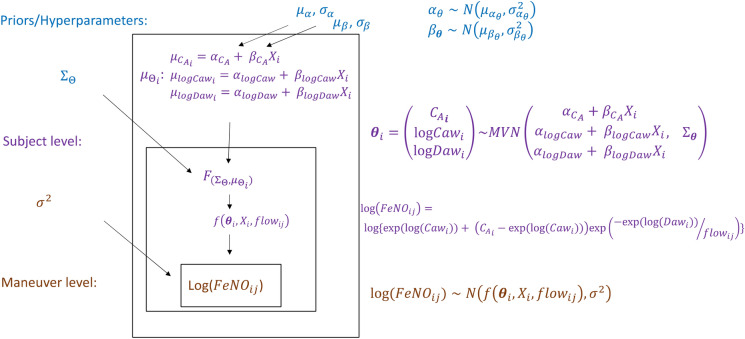
Hierarchical model structure relating FeNO measurements at multiple flow rates to NO parameters that are a function of a potential determinant X (e.g., air pollution).

In our unified approaches, we aim to model the measured outcome FeNO conditionally on both latent NO parameters and measured environmental factors using hierarchical modeling. Unified methods, in contrast to the TS methods, simultaneously estimate the NO parameters and their associations with the covariate ${X}_{i}$ in a single model. Below we present two unified methods, one based on the frequentist NLME approach and our novel method using a Bayesian approach:

1. The Unified Nonlinear Mixed Effect model (U-NLME): In U-NLME, rather than estimate separate Stage II models relating estimated NO parameters to ${X}_{i}$ as in TS-NLME, we incorporate ${X}_{i}$ into the mean function for each NO parameter in the NLME model (equations are conceptually similar to those in the U-HB, presented in Eqs. 5–7). Recall that NLME is recognized to have convergence issues and is sensitive to starting values^19,21^. Early work on the U-NLME indeed demonstrated problems with convergence^20^. However, for completeness we include U-NLME for comparison purposes.
2. The Unified Hierarchical Bayesian model (U-HB): The U-HB method can be described as a two-level hierarchical Bayesian model, with *j* indexing FeNO maneuvers nested in participant *i*, as displayed in Fig. 1.

### Two-level hierarchical Bayesian model (Fig. 1)

#### Level 1: Maneuver

Similar to Eq. (4), we assume that *log(FeNO)* for participant *i* at maneuver *j* is normally distributed:

5 $$\log \left( {{\text{FeNO}}_{ij} } \right) \sim N\left( {f\left( {\theta_{i} , X_{i} , flow_{ij} } \right),\sigma^{2} } \right)$$

with a mean function:

6 $$f\left( {\theta_{i} , X_{i} , flow_{ij} } \right) = \log \left( {\exp \left( {\log C_{aw_i} } \right) + \left( {C_{A_i} - \exp \left( {\log C_{aw_i} } \right)} \right) \times e^{{ - \exp \left( {\log D_{aw_i} } \right)/flow_{ij} }} } \right)$$

that depends on the 3-dimensional vector of participant-specific NO parameters ${{\varvec{\theta}}}_{i}=({{C}_{A}}_{i}, {{logC}_{aw}}_{i}, {{logD}_{aw}}_{i}){^{\prime}}$, ${X}_{i}$ (the role of ${X}_{i}$ becomes clear in Level 2), and flow rates. The variance (*σ*^2^) is assumed constant across participants and flow rates.

#### Level 2: Subject

We assumed the participant-level NO parameters are each a linear function of ${X}_{i}$:

7 $$\theta_{i} = \left[ {\begin{array}{*{20}c} {C_{A_i} } \\ {\log C_{aw_i} } \\ {\log D_{aw_i} } \\ \end{array} } \right] = \left[ {\begin{array}{*{20}c} {\alpha_{{C_{A} }} + \beta_{{C_{A} }} X_{i} + \epsilon_{{C_{A_i} }} } \\ {\alpha_{{\log C_{aw} }} + \beta_{{\log C_{aw} }} X_{i} + \epsilon_{{\log C_{aw_i} }} } \\ {\alpha_{{\log D_{aw} }} + \beta_{{\log D_{aw} }} X_{i} + \epsilon_{{\log D_{aw_i} }} } \\ \end{array} } \right]$$

where the vector ${{\varvec{\epsilon}}}_{i}=({\epsilon }_{{{C}_{A}}_{i}}, {\epsilon }_{{{logC}_{aw}}_{i}}, {\epsilon }_{{{logD}_{aw}}_{i}}){^{\prime}}$ of participant-level random effects is assumed to have a multivariate normal distribution with mean zero and variance–covariance matrix ${\Sigma }_{{\varvec{\theta}}}$. Based on the previous data analyses^38^, we assumed the joint distribution of the NO parameters can be reasonably modeled using a multivariate normal (MVN) distribution, and the equations above are equivalent to a formulation of: ${{\varvec{\theta}}}_{i}\sim MVN\left({{{\varvec{\mu}}}_{{\varvec{\theta}}}}_{i},{{\varvec{\Sigma}}}_{{\varvec{\theta}}}\right)$ where ${{{\varvec{\mu}}}_{{\varvec{\theta}}}}_{i}$ represents a mean vector with, for example, the first element equal to ${\alpha }_{{C}_{A}}+ {\beta }_{{C}_{A}}{X}_{i}$, and ${{\varvec{\Sigma}}}_{{\varvec{\theta}}}$ is the same variance–covariance matrix of NO parameters. Since the concentration of NO in the alveolar region should be non-negative, we imposed a non-negative constraint on ${C}_{A}$. We implemented our U-HB model using the Gibbs sampling program JAGS^39^. But JAGS can only specify univariate truncated normal distributions. To achieve this constraint in our multivariate context, we constructed the truncated MVN distribution into two steps. First, we sampled from a bivariate normal distribution for (${logC}_{aw}$, ${logD}_{aw}$), then we sampled from a zero-truncated normal distribution for ${C}_{A}$ with the conditional expectation and variance given (${logC}_{aw}$, ${logD}_{aw}$).

#### Prior distributions

We assumed the vectors of regression intercepts ($\boldsymbol{\alpha }$) and slopes (${\varvec{\beta}}$) in Eq. (7) each had independent normal prior distributions (with *I* indicating a square identity matrix):

8 $$\begin{array}{*{20}c} {{\varvec{\alpha}} \sim MVN\left( {{\varvec{\mu}}_{{\varvec{\alpha}}} , I\sigma_{\alpha }^{2} } \right)} \\ {{\varvec{\beta}} \sim MVN\left( {{\varvec{\mu}}_{{\varvec{\beta}}} , I\sigma_{\beta }^{2} } \right)} \\ \end{array}$$

where ${{\varvec{\mu}}}_{\boldsymbol{\alpha }}={\left({\mu }_{{\alpha }_{{C}_{A}}},{\mu }_{{\alpha }_{{logC}_{aw}}},{\mu }_{{\alpha }_{{logD}_{aw}}}\right)}^{^{\prime}}$= (2, 4.2, 2.5)${^{\prime}}$ and ${{\varvec{\mu}}}_{{\varvec{\beta}}}=({\mu }_{{\beta }_{{C}_{A}}}, {\mu }_{{\beta }_{{logC}_{aw}}}, {\mu }_{{\beta }_{{logD}_{aw}}}){^{\prime}}$= (0, 0, 0). Non-informative values were assumed for the variance hyperparameters ${\sigma }_{\alpha }^{2}$ $={\sigma }_{\beta }^{2}$= ${10}^{3}$. Similarly, the variance of the residuals (*σ*^2^) was assigned a non-informative *Inv-Gamma *(${10}^{-3}$, ${10}^{-3}$) prior distribution and the variance–covariance matrix of NO parameters (${{\varvec{\Sigma}}}_{{\varvec{\theta}}}$) was assigned a non-informative inverse-Wishart prior distribution.

#### Calculation of posterior via MCMC

Taking a Bayesian perspective, the general posterior distribution can be written as:

9 $$\begin{gathered} p(\mu_{\alpha } ,\sigma^{2}_{\alpha } ,\mu_{\beta } ,\sigma^{2}_{\beta } ,\Sigma_{\Theta } |\log FeNO_{ij} ,X_{i} ,flow_{ij} ) \propto p(\log FeNO_{ij} |f\left( {{{\varvec{\uptheta}}}_{i} ,flow_{ij} } \right),\sigma^{2} ) \hfill \\ p({{\varvec{\uptheta}}}_{i} |\mu_{{{{\varvec{\uptheta}}}_{i} }} ,{\Sigma }_{{{\varvec{\uptheta}}}} )p(\mu_{{{{\varvec{\uptheta}}}_{i} }} |{\varvec{\alpha}},{\varvec{\beta}},X_{i} )p({\varvec{\alpha}}|\mu_{{\varvec{\alpha}}} ,\sigma^{2}_{{\varvec{\alpha}}} )p({\varvec{\beta}}|\mu_{\beta } ,\sigma^{2}_{\beta } )p\left( {\sigma^{2} } \right) \hfill \\ \end{gathered}$$

This density cannot be directly calculated. Instead, we rely on MCMC methods to provide samples from the density. Specifically, we implemented Gibbs sampling using JAGS, taking advantage of normal conjugate distributions for this hierarchical model. While convergence is sometimes non-trivial to obtain, Gibbs sampling generally had good performance in the models considered here.

To conduct U-HB analyses, we used three parallel MCMC chains in JAGS. Once the Gelman-Rubin $\widehat{R}$ convergence criteria^40^ reached ≤ 1.1, we drew 12,000 additional samples and checked that the $\widehat{R}$ remained ≤ 1.1 (second checkpoint). If so, we used those 12,000 samples to construct the posterior distributions of the parameters. Otherwise, we continued sampling as long as necessary to satisfy the convergence criteria.

### Simulation study

We conducted an extensive simulation study to compare the performance of the five methods. To produce realistic synthetic data, we modelled our data generation scenarios on the CHS. Specifically, we simulated data for 1000 individuals and assumed each individual performed 8 FeNO maneuvers, two at each of four flow rates: 30, 50, 100, and 300 ml/s. These particular flow rates were selected for three reasons: (1) they match the flow restrictors provided with the FeNO sampling instrument, (2) they are within a reasonable range of flows for schoolchildren, and (3) optimal flow rate sampling design has been studied in detail previously, and studies with these flow rates balanced theoretical performance with feasibly^36^. Based on previously described distributions in the CHS^38^, we assumed NO parameters had a multivariate normal distribution with an additional non-negative constraint for ${C}_{A}$ in data generation step (the randomly sampled vector of NO parameters was discarded if it had a negative ${C}_{A}$ value). The variance–covariance matrix was set to be similar to values observed in preliminary analyses of CHS data:

10 $${\Sigma }_{{\Theta }} = \left( {\begin{array}{*{20}c} {0.44} & {0.13} & { - 0.14} \\ {0.13} & {0.62} & { - 0.15} \\ { - 0.14} & { - 0.15} & {0.36} \\ \end{array} } \right)$$

We also assumed that a standard normal covariate X had a potentially different linear relationship with each NO parameter. In all data generating scenarios, we set the fixed effect intercepts (mean NO parameters for participants with mean X) to mirror those used in previous statistical methods work on FeNO in the CHS (i.e., 1, 3.5, and 2.5 for *C*_*A*_, *logC*_*aw*_ and *logD*_*aw*_ respectively). Our primary focus was on the associations of X with NO parameters (${\beta }_{{C}_{A}}$, ${\beta }_{{logC}_{aw}}$, and ${\beta }_{{logD}_{aw}}$).

We considered seven different scenarios, in which one or more of the $\beta$’s varied (ranging from 0 to 0.2, with a step size of 0.02) as summarized in Table 1. In Scenario 1, the magnitudes of the associations between X and each NO parameters were assumed to be equal (${\beta }_{{C}_{A}}$= ${\beta }_{{logC}_{aw}}$= ${\beta }_{{logD}_{aw}}$) and all $\beta$’s varied together. In Scenarios 2–4, only one of the NO parameters was associated with X; in Scenarios 5–7, only one NO parameter was not associated with X. The scenarios where at least one association is truly zero permit estimation of Type I error rates. For each setting of each scenario, we conducted 1000 replicates. Using these seven scenarios, we compared the five methods in terms of the following criteria. Bias was calculated as (estimate–true value) and relative bias as (estimate–true value)/true value. Considering 95% confidence intervals or credible intervals (henceforth called 95% CI for simplicity), we calculated their length and coverage. Power was defined as the proportion of the 95% CIs that did not include 0 when the true value was not 0. We used Scenario 1 for primary results, where all NO parameters were assumed to be equally affected by X. We used Scenarios 5–7 for primary results on type I error rates, which we define as the proportion of 95% CIs that did not include 0 when the true value was 0. All results were presented only for simulated datasets on which all methods satisfied convergence criteria.

**Table 1:** Seven scenarios considered in the simulation study, where the relation of X to each NO parameter (${\beta }_{{C}_{A}}$, ${\beta }_{{logC}_{aw}}$, ${\beta }_{{logD}_{aw}}$) varied from 0 to 0.2, with a step size of 0.02.

	${\beta }_{{C}_{A}}$	${\beta }_{{logC}_{aw}}$	${\beta }_{{logD}_{aw}}$
Scenario 1*	0–0.2	0–0.2	0–0.2
Scenario 2	0.02–0.2	0^†^	0
Scenario 3	0	0.02–0.2	0
Scenario 4	0	0	0.02–0.2
Scenario 5*	0	0.02–0.2	0.02–0.2
Scenario 6*	0.02–0.2	0	0.02–0.2
Scenario 7*	0.02–0.2	0.02–0.2	0

*In Scenarios 1 and 5–7, the non-zero *β* values are identical (e.g., the first three settings of Scenario 1 have ${\beta }_{{C}_{A}}$= ${\beta }_{{logC}_{aw}}$= ${\beta }_{{logD}_{aw}}$= 0, ${\beta }_{{C}_{A}}$= ${\beta }_{{logC}_{aw}}$= ${\beta }_{{logD}_{aw}}$= 0.02, ${\beta }_{{C}_{A}}$= ${\beta }_{{logC}_{aw}}$= ${\beta }_{{logD}_{aw}}$= 0.04).

^†^Cells marked 0 indicate that X had no effect on the corresponding NO parameter.

In total, we generated 71 sets of simulated datasets (7 scenarios with 10 values each of the varying parameters, as well as one dataset with all $\beta$’s = 0), each replicated 1,000 times. Analyses were conducted using the clusters at the University of Southern California’s High-Performance Computing Center using R version 3.5 and JAGS 4.0. The median time needed to produce estimates for 5 models (TS-NLS, TS-HMA, TS-NLME, U-NLME, U-HB) for each dataset was 6 h.

### CHS data analysis

In a previous publication using data from CHS participants^38^, we investigated the association between traffic-related air pollution (TRAP) and NO parameters. Briefly, we conducted a cross-sectional analysis of multiple flow FeNO measured in 1635 schoolchildren ages 12–15, using TS-NLME and TS-HMA. By design, CHS participants had ~ 9 FeNO maneuvers each (3 at 50 ml/s, 2 each at 30, 100, and 300 ml/s flow rates). Exposure to TRAP in the indoor schoolroom microenvironment at the time of the FeNO test was assessed using approximately concurrent room air measurements of NO (ppb). In this paper, we re-analyzed these data using both “unadjusted” models (with TRAP exposure as the only covariate) and minimally “adjusted” models (with TRAP exposure plus adjustment for: sex, age, asthma). The original publication adjusted for additional potential confounders or study design variables (race/ethnicity, rhinitis history, use of asthma medications in the past 12 months, secondhand tobacco smoke exposure, parental education, time of day of FeNO test, FeNO analyzer, and CHS community), but results from minimally adjusted models were similar to those of the fully adjusted model^38^. Note that the original publication used the alternative *J’*_*aw*_ parameterization of the two-compartment model:

11 $$\log \left( {{\text{FeNO}}} \right) = \log (\exp (\log J_{{aw}}^{\prime } - \log D_{{aw}} ) + \left( {C_{A} - \exp \left( {\log J_{{aw}}^{\prime } - \log D_{{aw}} } \right)} \right)*\exp ( - \exp (\log D_{{aw}} )/flow) + \varepsilon$$

whereas in this paper we used the “*C*_*aw*_” parameterization (Eq. 2) due to the direct estimation of the more interpretable airway wall concentration parameter. Thus, here we fit TS-NLME models to CHS data with both the “$J_{aw}^{^{\prime}}$” and “*C*_*aw*_” parameterization to facilitate direct comparison with the previously published work.

## Results

### Simulation study

#### Computational time and convergence

TS-NLS and TS-HMA models always converged in Stage II and their run times were typically less than 1 min (Supplementary Fig. 1), but they failed to estimate NO parameters in Stage I for some participants due to small sample size. In average, 31.6% of the Stage I models failed to converge for TS-NLS while it was < 1% for TS-HMA. Hence these participants were left out of the Stage II model. TS-NLME models had a convergence rate of 90% (average time: ~ 10 min) while U-NLME had a convergence rate of only 70% (average time: ~ 20 min). For U-HB models, the median time to achieve $\widehat{R}$ < 1.1 was 5.5 h (~ 90% of models converged within 16 h and 1% never converged in the 48 h allowed). For the rest of this paper, to aid comparison, we only show results obtained from the subset of datasets for which all methods satisfied converged criteria. Results for all datasets are shown in Supplementary Sect. 5.

**Figure 2:**
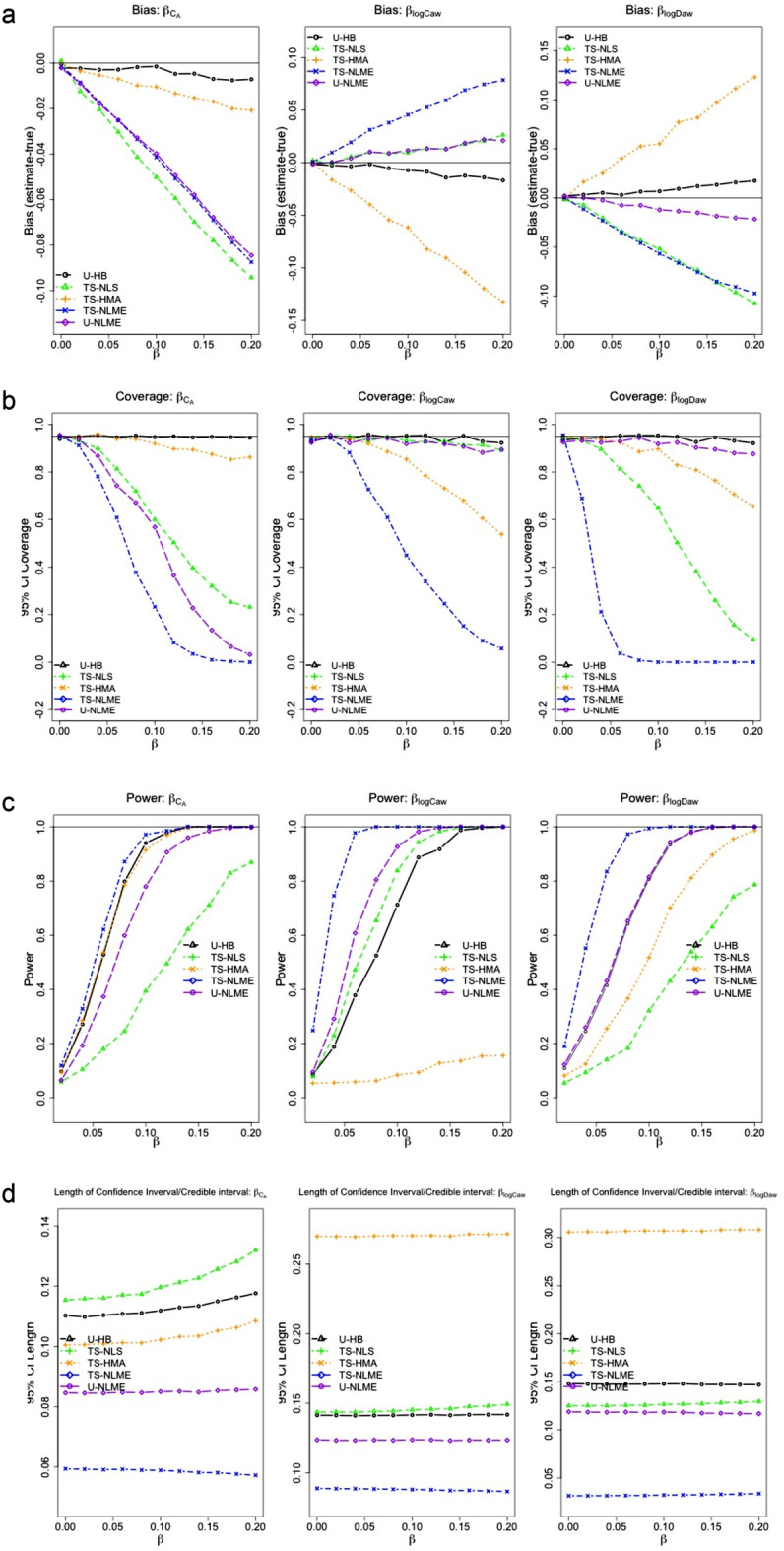
Relative bias (**a**), coverage (**b**), power (**c**) and CI length (**d**) of the selected estimation methods from Scenario 1 of the simulation study (${\beta }_{{C}_{A}}$= ${\beta }_{{logC}_{aw}}$= ${\beta }_{{logD}_{aw}}$).

*Bias*. As shown in Fig. 2a (Scenario 1), U-HB consistently showed the smallest bias. For other methods, the directions and relative magnitudes of the bias varied across NO parameters. Trends in bias were consistent across the range of $\beta$, so we report average relative bias. All methods produced negatively biased estimates of ${\beta }_{{C}_{A}}$, with the average relative bias being the smallest for U-HB (− 4.1%) and much larger for the other methods (approximately − 12.3% for TS-HMA and − 50.4% for TS-NLS, − 42.6% for TS-NLME and − 42.3% for U-NLME). For ${\beta }_{{logC}_{aw}}$, average relative bias was negative for U-HB (− 7.7%) and TS-HMA (− 67.6%) but positive for the other methods (11.1% for U-NLME, 11.4% for TS-NLS, and 45.1% for TS-NLME). Conversely, for ${\beta }_{{logD}_{aw}}$, the average relative bias was positive for U-HB (8.8%) and TS-HMA (64.1%) but negative for the other methods (− 10.2% for U-NLME, -53.4 for TS-NLS, and -55.2% for TS-NLME). For Scenarios 2–7 (Supplementary Figs. 2.2–2.7), U-HB also had smaller bias than all other methods. In the secondary analyses, constrained TS-NLS had smaller bias than the standard TS-NLS for estimating ${C}_{A}$, but, it considerably underestimated *logC*_*aw*_. Inverse-variance weighted TS-NLS had even worse performance (results not shown). Thus, we only report standard TS-NLS results.

#### CI coverage and length

As shown in Fig. 2b and d (Scenario 1), U-HB was the only method to produce CIs with appropriate coverage (~ 95%) for associations with all NO parameters (${\beta }_{{C}_{A}}$, ${\beta }_{{logC}_{aw}}$, ${\beta }_{{logD}_{aw}}$). TS-NLME consistently had the worst coverage while its one-step analog, U-NLME, performed much better for ${\beta }_{{logC}_{aw}}$ and ${\beta }_{{logD}_{aw}}$ but only moderately better for ${\beta }_{{C}_{A}}$. To further understand this difference in TS-NLME vs U-NLME coverage, we note that the CIs for TS-NLME were very narrow (the narrowest of all methods) and TS-NLME estimates were considerably more biased than for U-NLME (except for ${\beta }_{{C}_{A}}$, where the bias was similar for the two methods). TS-NLS had poor coverage for ${\beta }_{{C}_{A}}$ (due to large bias, despite wide CI) and ${\beta }_{{logD}_{aw}}$ (due to large bias). TS-HMA had the widest CIs for ${\beta }_{{log}_{Caw}}$ and ${\beta }_{{log}_{Daw}}$ but the bias for these parameters was also large, reducing the CIs’ coverage. For Scenarios 2–7, results were similar (Supplementary Figs. 2.2–2.7).

#### Type I error

**Figure 3:**
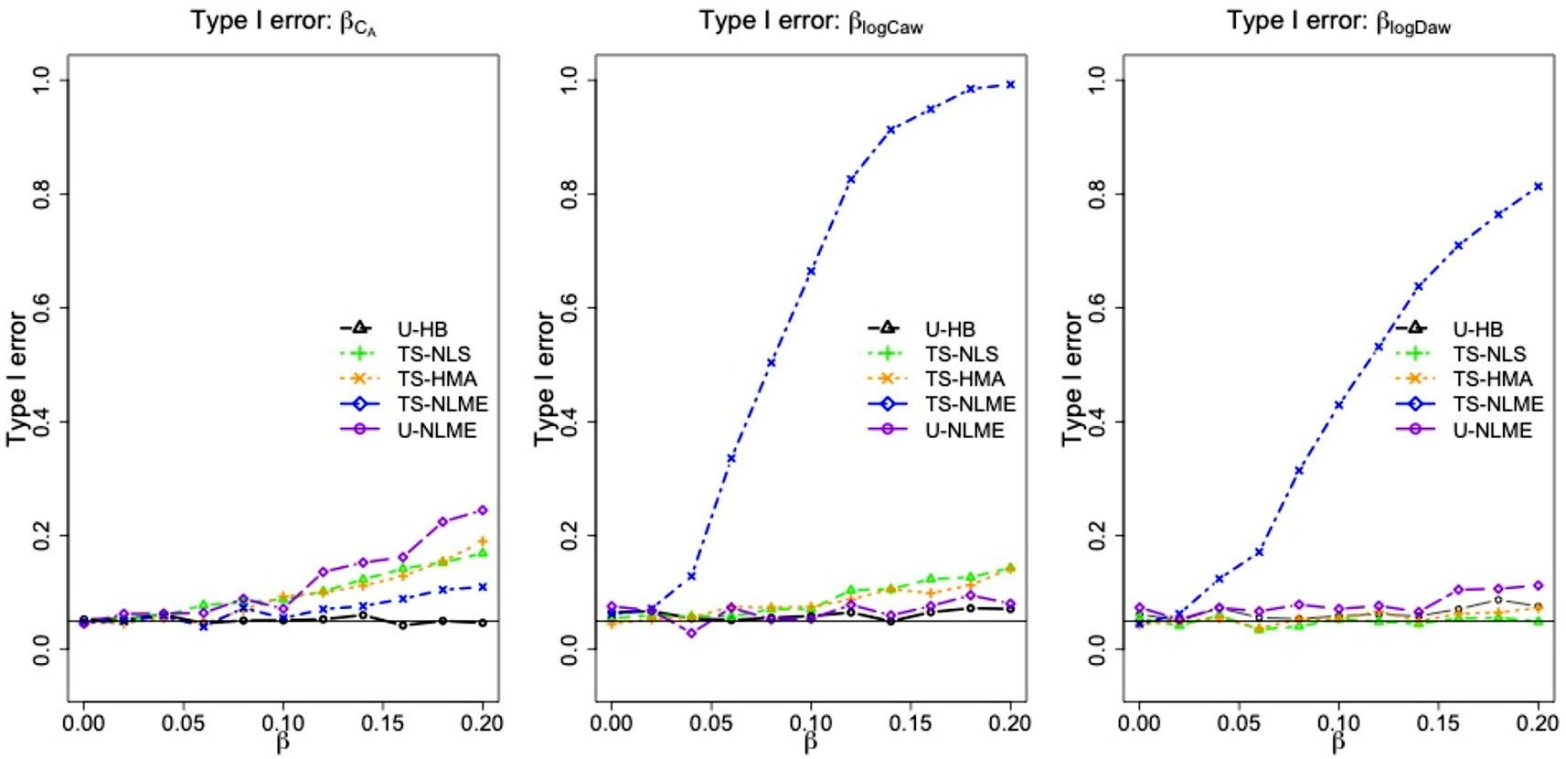
Type I error* of the selected estimation methods from the simulation study. * With the type I error calculated for ${\beta }_{{C}_{A}}$ using Scenario 5 with ${\beta }_{{C}_{A}}$= 0, ${\beta }_{{logC}_{aw}}$= ${\beta }_{{logD}_{aw}}$; for ${\beta }_{{logC}_{aw}}$ using Scenario 6 with ${\beta }_{{C}_{A}}$= ${\beta }_{{logD}_{aw}}$, ${\beta }_{{logC}_{aw}}=0$; and for ${\beta }_{{logD}_{aw}}$ using Scenario 7 with ${\beta }_{{C}_{A}}$= ${\beta }_{{logC}_{aw}}$, ${\beta }_{{logD}_{aw}}=0$).

Figure 3 shows type I error rates from Scenarios 5, 6 and 7, all of which had only one NO parameter not affected by the covariate X (e.g., type I error for ${\beta }_{{C}_{A}}$ was obtained from Scenario 5 where ${\beta }_{{C}_{A}}=0$ while ${\beta }_{{logC}_{aw}}={\beta }_{{logD}_{aw}}$ varied). Results of Scenarios 2–4, in which only one NO parameter association was non-zero, are shown in Supplementary Figs. 2.2–2.4. Of all the methods, U-HB generally had the lowest type I error rates, with values of ~ 0.05 for ${\beta }_{{C}_{A}}$, ${\beta }_{{logC}_{aw}}$, and ${\beta }_{{logD}_{aw}}$ but that increased slightly as the effect of X on the other NO parameters increased. As might be expected from the earlier analyses of bias and CI coverage/length, the other methods performed less well. TS-NLME had poor type I errors for ${\beta }_{{logC}_{aw}}$ and ${\beta }_{{logD}_{aw}}$, and the type I errors increased dramatically as the effect of X increased. U-NLME had the highest type I error for ${\beta }_{{C}_{A}}$, and the type I error increased more modestly as the effect of X increased. TS-NLS and TS-HMA had similar patterns in type I error rates, which were inflated slightly for ${\beta }_{{C}_{A}}$ and ${\beta }_{{logC}_{aw}}$ but ~ 0.05 for ${\beta }_{{logD}_{aw}}$.

#### Power

As shown in Fig. 2c (Scenario 1), power results were complex. U-HB had power > 0.99 for effect sizes of 0.12 for ${\beta }_{{C}_{A}}$, 0.18 for ${\beta }_{{log}_{Caw}}$, and 0.16 for ${\beta }_{{log}_{Daw}}$. Although U-HB never had the best power relative to the other methods, the apparently good power curves of some methods require careful interpretation. TS-NLME appeared to have the best power across all NO parameters, but this was due to its narrow confidence intervals and came at the cost of inflated type-I error rates (e.g., Fig. 2c for Scenario 1 when ${\beta }_{{C}_{A}}$= ${\beta }_{{logC}_{aw}}$= ${\beta }_{{logD}_{aw}}$= 0). TS-HMA had a similar power curve to U-HB for ${\beta }_{{C}_{A}}$ (the parameter for which TS-HMA had the least bias), but TS-HMA had low power curves for ${\beta }_{{log}_{Caw}}$ and ${\beta }_{{log}_{Daw}}$ (parameters for which TS-HMA had displayed considerable bias and wide CI). Relative to the other methods, U-NLME power was high for ${\beta }_{{log}_{Caw}}$ and ${\beta }_{{log}_{Daw}}$, but poor for ${\beta }_{{C}_{A}}$ (the parameter for which U-NLME had considerable bias). TS-NLS had poor power for ${\beta }_{{C}_{A}}$ and ${\beta }_{{log}_{Daw}}$ but better power for ${\beta }_{{log}_{Caw}}$(the parameter for which TS-NLS had the least bias).

#### Overall summary of simulation study results

Across our simulation study scenarios, U-HB had good statistical properties and consistently outperformed U-NLME and the three TS approaches. The other unified approach, U-NLME, performed well for ${\beta }_{{logC}_{aw}}$ and ${\beta }_{{logD}_{aw}}$, but not for ${\beta }_{{C}_{A}}$ (biased, poor coverage, high type I error) and suffered from serious convergence issues, failing to converge for 30.2% of simulated datasets (Supplementary Fig. 1). The worst bias was observed for TS methods. Interestingly, TS-HMA had a different direction of bias compared to TS-NLS, TS-NLME and U-NLME for ${\beta }_{{logC}_{aw}}$ and ${\beta }_{{logD}_{aw}}$. The correlation between NO parameters had an influence on bias across all methods. We assumed a positive correlation between *C*_*A*_ and *logC*_*aw*_, a negative correlation between *C*_*A*_ and *logD*_*aw*_*,* and a negative correlation between *logC*_*aw*_ and *logD*_*aw*_ to mimic the distributions previously observed in CHS participants. Neither the magnitude nor the direction of the estimated relative bias for ${\beta }_{{C}_{A}}$ from all the models were affected by the values of ${\beta }_{{logC}_{aw}}$ or ${\beta }_{{logD}_{aw}}$ (comparing bias figures for Scenario 1 to Scenarios 2, 6 and 7, recall all Scenarios are included in Supplementary Figs. 2.1–2.7). But when estimating ${\beta }_{{logC}_{aw}}$ and ${\beta }_{{logD}_{aw}}$, the relative bias changed both in magnitude and direction, except for U-HB (comparing bias figures for Scenario 1 to Scenarios 3, 5 and 7; comparing Scenario 1 to Scenarios 4, 5 and 7). We focused the simulation study results on $\beta$’s, the association between X and NO parameters, as our main interest. Results for $\alpha$’s, the population mean NO parameters when X = 0, are presented in Supplementary Figs. 3.1–3.7.

### CHS data analysis

**Figure 4:**
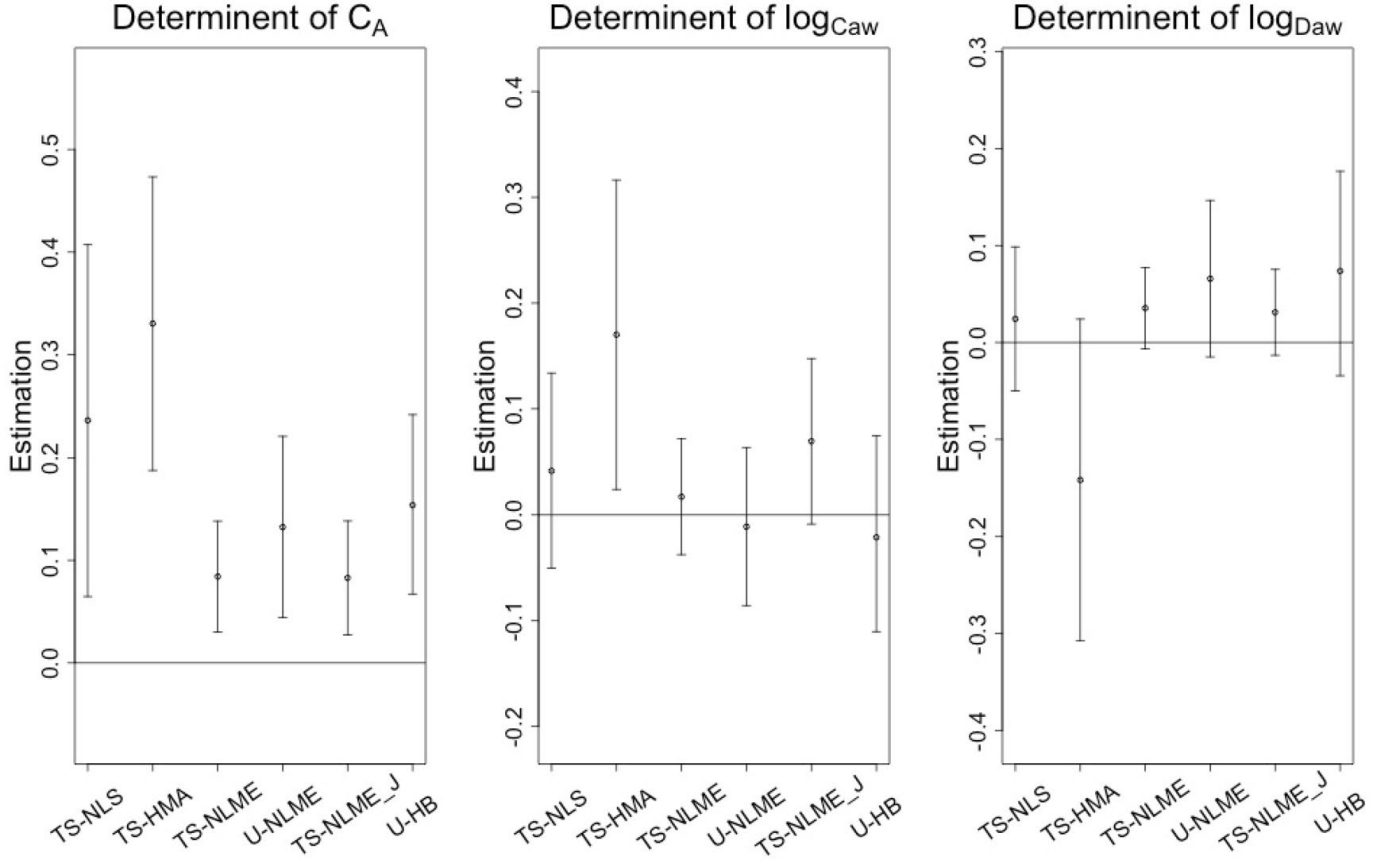
Analysis of CHS data: estimated associations of traffic-related air pollution with *C*_*A*_, *logC*_*aw*_, and *logD*_*aw*_ ($\widehat{\beta }$ and 95% CI) using the selected methods, *with no adjustments for covariates. *TS-NLME ($J_{aw}^{^{\prime}}$) is the previously published model using a $J_{aw}^{^{\prime}}$ parameterization of TS-NLME.

As shown in Fig. 4, we confirmed the previously published result (using the *J’*_*aw*_ parameterization TS-NLME)^38^ of a statistically significant positive association between TRAP and alveolar NO (${\beta }_{{C_{A}}}$) using all estimation methods. Results were quite similar with and without adjustment for age, sex, and asthma status (Table 2). Using U-HB, we found that a 10 ppb higher TRAP concentration was associated with a 0.15 (95% CI: 0.07, 0.24) ppb increase in C_A_, after adjusting for sex, age, and asthma. This U-HB estimated ${\beta }_{{C_{A}}}$ was nearly twice the size of the analogous estimate from TS-NLME (0.08, 95% CI: 0.03, 0.14 from both parameterizations). As in the previous publication, there was little evidence for effects of TRAP on airway wall inflammation (${\beta }_{{logC_{aw}}}\;{\hbox{and}}\; {\beta }_{{logD_{aw}}}$). Based on the CHS findings, Scenario 2 of the simulation study (${\beta }_{{C_{A}}}$ varies, ${\beta }_{{logC_{aw}}} = 0,\, {\beta }_{{logD_{aw}}} = 0$, Supplemental Fig. 2.2) might be a closer comparator to the CHS data than Scenario 1. The finding in the CHS data that the estimated ${\beta }_{{C_{A}}}$ was larger for U-HB than for TS-NLME agrees with results from both simulation Scenarios 1 and 2, where U-HB had a modest negative bias for ${\beta }_{{C_{A}}}$ compared to the more considerable negative bias for TS-NLME. While we were motivated by the problem of estimating TRAP associations with NO parameters (Stage II), it is interesting to note that all methods produce participant-level NO parameter estimates (Stage I) and these estimates were most similar when comparing U-HB to TS-NLME and U-NLME (Supplemental Figs. 4.1–4.3). However, TS-NLS and TS-HMA failed to estimate NO parameters in Stage I for subsets of participants due to small sample size of flow rate (31.6% for TS-NLS and <1% for TS-HMA in average).

**Table 2:** Analysis of CHS data: estimated associations of a 10ppb increase in traffic-related air pollution with *C*_*A*_, *logC*_*aw*_, and *logD*_*aw*_ ($\widehat{\beta }$ and 95% CI) using the selected methods, without and with adjustments for covariates (age, sex, asthma).

Estimation method	Model	Estimated ${{\varvec{\beta}}}_{{{\varvec{C}}}_{{\varvec{A}}}}$ (95% CI)	Estimated ${{\varvec{\beta}}}_{{{\varvec{l}}{\varvec{o}}{\varvec{g}}{\varvec{C}}}_{{\varvec{a}}{\varvec{w}}}}$ (95% CI)	Estimated ${{\varvec{\beta}}}_{{{\varvec{l}}{\varvec{o}}{\varvec{g}}{\varvec{D}}}_{{\varvec{a}}{\varvec{w}}}}$ (95% CI)
TS-NLS	Unadjusted	0.24 (0.06, 0.41)**	0.04 (− 0.05, 0.13)	0.02 (− 0.05, 0.10)
	Adjusted	0.23 (0.06, 0.40)**	0.04 (− 0.05, 0.13)	0.03 (− 0.05, 0.10)
TS-HMA	Unadjusted	0.33 (0.19, 0.47)**	0.17 (0.02, 0.32)*	− 0.14 (− 0.31, 0.02)
	Adjusted	0.32 (0.18,0.46)**	0.16 (0.02, 0.31)*	− 0.14 (− 0.31 0.02)
TS-NLME	Unadjusted	0.08 (0.03, 0.14)**	0.02 (− 0.04, 0.07)	0.04 (− 0.01, 0.08)
	Adjusted	0.08 (0.03, 0.14)**	0.01 (− 0.04, 0.07)	0.03 (− 0.01, 0.08)
TS-NLME ($J_{aw}^{^{\prime}}$)^†^	Unadjusted	0.08 (0.03, 0.14)**	0.07 (− 0.01, 0.15)	0.03 (0.01, 0.08)
	Adjusted	0.08 (0.03, 0.14)**	0.07 (− 0.01, 0.14)	0.03 (− 0.01, 0.07)
U-NLME	Unadjusted	0.13 (0.04, 0.22)**	− 0.01 (− 0.09, 0.06)	0.07 (− 0.01, 0.15)
	Adjusted	0.13 (0.05, 0.22)**	− 0.03 (− 0.09, 0.06)	0.07 (− 0.01, 0.15)
U-HB	Unadjusted	0.15 (0.07, 0.24)	− 0.02 (− 0.11, 0.07)	0.07 (− 0.03, 0.18)
	Adjusted	0.15 (0.07, 0.24)	− 0.02 (− 0.12, 0.07)	0.07 (− 0.04, 0.18)

^†^Previously published model: $J_{aw}^{^{\prime}}$ parameterization of TS-NLME.

*P  <  0.05, **P  <  0.01, ***P  <  0.001.

## Discussion

In this paper, we performed an extensive simulation study to evaluate the finite sample performance of a variety of statistical methods used to estimate the associations of covariates with NO parameters from the steady-state two compartment model of lower respiratory tract NO. One of these methods was a novel Unified Hierarchical Bayesian (U-HB) model which simultaneously estimates participant-level NO parameters and their associations with covariates. In the simulation studies, U-HB outperformed four other methods: three two-stage methods and one unified method, all implemented using frequentist approaches. When applying U-HB to the motivating data example—investigating associations of traffic-related pollution exposure with NO parameters in a cross-sectional sample of southern California schoolchildren—we confirmed the previously published positive association of traffic with alveolar inflammation (*C*_*A*_). However, the estimated association was nearly two times larger (0.15 vs 0.08) using U-HB as compared to the two-stage method (TS-NLME) applied in the previous publication. This result was consistent with the simulation study finding that estimates of ${\beta }_{{C_{A}}}$ tended to be higher (less negative bias) in U-HB than in TS-NLME.

Numerous applied publications use the two-stage approach, with NO parameters estimated in Stage I and then treated as observed outcomes in Stage II analyses relating NO parameters to covariates. Our paper is, to our knowledge, the first evaluation of statistical methods used to estimate the associations of covariates with NO parameters. A number of publications have previously studied statistical methods for estimating participant-level NO parameters (i.e., only Stage I)^9,13,19^. In our own previous comparison of Stage I methods, we found that NLS outperformed linear, quadratic, and third order approximations (i.e., HMA), especially in terms of reduced bias for estimates of ${\mathrm{D}}_{aw}NO$ and more appropriate CI coverage for all NO parameters^19^. In this paper where we compare TS methods, we found that TS-NLS also generally outperformed TS-HMA. The direction of bias for TS-NLS and TS-NLME were always the same, while TS-HMA usually differed from the other methods. TS-HMA also had the widest CI while TS-NLME had the shortest CI (resulting in high power but low coverage). The worst bias was observed for TS methods, which we suspect was a consequence of not propagating uncertainty in the second stage. U-NLME, which had the second best performance after U-HB, had serious convergence issues, as we had reported in our early work on U-NLME^20^.

This paper has several strengths. First, our novel U-HB model takes a unified approach, which combines the estimation of NO parameters and their associations with covariates into a single step. This allows for the propagation of uncertainty across stages and avoids the exclusion of some participants in two-stage models due to failure to estimate their Stage I model. Second, we used a Bayesian approach for U-HB which allowed us to: clearly specify a hierarchical model structure, fully characterize the posterior distributions of the parameters of interest, and to have the flexibility to use diffuse priors (as we do here) or more informative priors if appropriate. Unlike TS-NLS or TS-NLME, U-HB does not use inferential approaches that rely on large sample size/normality assumptions that might be violated with multiple flow FeNO datasets. Third, we used the *C*_*aw*_ parameterization of the 2CM (Eq. 1) for all the methods presented in the paper since it directly estimates what we believe to be a more interpretable NO parameter—the airway wall concentration of NO (*C*_*aw*_, ppb)—rather than $J_{aw}^{^{\prime}}$ (the airway wall tissue diffusion capacity, pL·s^−1^·ppb^−1^). Based on CHS data analysis (Supplementary Fig. 4.3), had we used the $J_{aw}^{^{\prime}}$ parameterization, our results would have been similar to what we observed using the *C*_*aw*_ parameterization. An additional advantage of U-HB is that, since it is implemented using MCMC, we can fully characterize the posterior of $J_{aw}^{^{\prime}}$ from a *C*_*aw*_ parameterization model by simply calculating exp (*log C*_*aw*_)/exp (*log D*_*aw*_) at each MCMC iteration. Fourth, we compared U-HB to four alternative methods, which spanned the range applied in practice to study the associations of covariates with NO parameters. We did exclude one common two-stage method (“TS-linT”) in which Stage I is a simple linear regression for each participant, based on the linear approximation proposed by Tsoukias et al.^41^. We excluded TS-linT because its simplified assumption of the approximation cannot estimate *C*_*aw*_ (it only estimates $J_{aw}^{^{\prime}}$ and *C*_*A*_). Fifth, we performed an extensive set of simulation studies across a range of effect sizes, which were of particular concern in situations where the covariate is related to only one (or two) of the three correlated NO parameters. We examined not only bias and CI length but also power and type I error rates in those scenarios to understand how each model behaved. Finally, we implemented U-HB via Gibbs sampling in readily available software (the R interface to JAGS). This implementation of the U-HB worked well across simulation scenarios (especially compared to other methods) and converged considerably better than the NLME methods.

Limitations of our work include the following three issues. First, the current implementation of U-HB in JAGS is computationally intensive and requires hours to fit instead of seconds (TS-NLS, TS-HMA), minutes (TS-NLME) or tens of minutes (U-NLME). To overcome this challenge in the exploratory or interactive model-building stage of an applied data analysis, an analyst may wish to use a two-stage version of HB. One can run U-HB with no covariates to obtain participant-level NO parameters in Stage 1, and then in Stage 2, run the usual separate linear regression models relating NO parameters to covariates. Penultimate and final models could then be estimated with U-HB. Running the final analyses using U-HB, over the course of several hours, is a minimal commitment of resources compared to the months or years that most studies have devoted to planning and data collection. Future work may consider alternative MCMC methods, with the goal of reducing run time. Second, our simulation studies though extensive, did not cover all possible scenarios. Here we generated data using magnitudes of association and NO parameter distributions similar to those observed in the CHS data, under a single covariate scenario. However, U-HB can be readily applied to scenarios with multiple covariates or even interactions, with some additional computational cost. For example using CHS data, U-HB converged after 4.8 h for the model with only one covariate, but 9.2 h for a model with two covariates and 9.5 h for a model with four covariates. Third, we used the conservative approach of only comparing methods using datasets for which all the methods converged. If we included all datasets (dropping a dataset for a method only if that method failed to converge on that dataset), the relative performance the methods was similar (Supplemental Fig. 5).

In summary, we have performed the first evaluation of statistical methods used to estimate the associations of covariates with NO parameters. We found the best performance using a novel unified estimation method (U-HB), which requires longer computation time (hours rather than minutes) but which can be readily implemented using standard statistical software.

## Software

An example dataset and R code for implementing all 5 methods are available on Github https://github.com/USCbiostats/FeNO_UHB.

## Supplementary Information

Supplementary Information 1.

## Data Availability

Due to the limitations in the original consent forms and HIPAA requirements, the data from the CHS cannot be freely available in the manuscript, supplemental files, or in a public repository. However, we are committed to sharing the data and results acquired as part of this study. The CHS has a process in place for data sharing that involves approval of proposals by a Data Sharing Committee. Investigators who want access to data will be required to submit a research protocol, which will be reviewed by the CHS Health Data Release Committee and the USC IRB. Please send requests to access this dataset to Dr. Frank Gilliland (gillilan@usc.edu).

## List of symbols

NO parameters: *C*_*A*_ *, logC*_*aw*_ *, logD*_*aw*_
*C*_*A*_: Concentration of NO in the alveolar region, (ppb)
*C*_*aw*_: Concentration of NO in the airway tissue, (ppb)
*D*_*aw*_: Airway tissue diffusion capacity, pL·s^−1^·ppb^−1^
$J_{aw}^{{\prime}}$: Maximum flux of NO in the airway
FeNO: Fractional concentration of exhaled nitric oxide, ppb
NLME: Nonlinear mixed effect model
HMA: Högman and Merilӓinen Algorithm
