# Supplementary material for: Hierarchical Bayesian estimation of covariate effects on airway and alveolar nitric oxide
Source: Sci Rep. 2021 Aug 25;11:17180. doi: 10.1038/s41598-021-96176-z (PMC8387480; doi:10.1038/s41598-021-96176-z)
Supplement: Supplementary file 1 — Supplementary Information 1. [file 41598_2021_96176_MOESM1_ESM.docx]

**Online supplement for:**

Hierarchical Bayesian estimation of covariate effects on airway and alveolar nitric oxide

**Table of Contents**

[**Section 1: Convergence failure rates in the simulation study** 2](#_Toc39495986)

[**Section 2: Bias, Coverage, Power and Confidence Interval Length figures for** $\boldsymbol{\beta}$**, by scenario** 3](#_Toc39495987)

[**Section 3: Bias, Coverage, Power and Confidence Interval Length figures for** $\boldsymbol{\alpha}$**, by scenario.** 10](#_Toc39495988)

[**Section 4. Additional details on CHS results** 17](#_Toc39495989)

[**Section 5. Simulation study results using all available estimates (not limited to datasets for which all models converged).** 20](#_Toc39495990)

# **Section 1: Convergence failure rates in the simulation study**

**Supplementary Figure 1:** Rates of convergence failure in the simulated datasets, by method (denoted by line type) by scenario (denoted by line color, see paper for definitions), as a function of effect size, using simulated datasets.


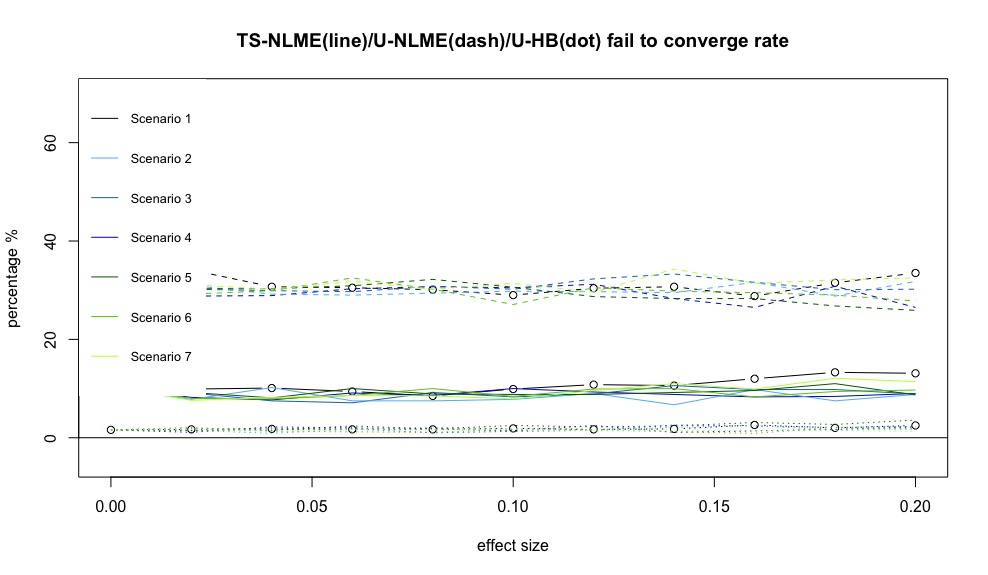


There was no trend in convergence failure rate by Scenario or effect size, so we summarized those rates using the average across Scenarios and effect sizes. There were notable difference in these rates across methods: 9% for TS-NLME, 30% for U-NLME and only 2% for U-HB.

# **Section 2: Bias, Coverage, Power and Confidence Interval Length figures for** $\boldsymbol{\beta}$**, by scenario**

In this section, we provide full results for our simulation study for all seven Scenarios. Results for Scenario 1 were included in the main body of the manuscript, but we repeat them below for completeness.

**Supplementary Figure 2.1**. Relative bias (a), coverage (b), and power (c) CI length (d) of the selected estimation methods from Scenario 1 of the simulation study ($\beta_{C_{A}}$= $\beta_{{logC}_{aw}}$= $\beta_{{logD}_{aw}}$).
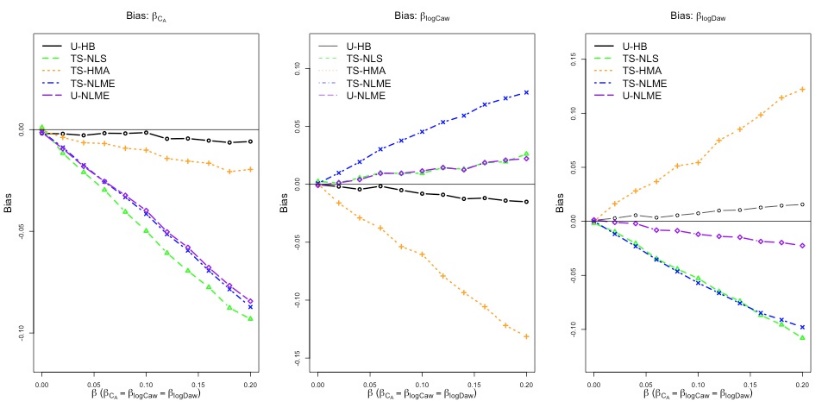

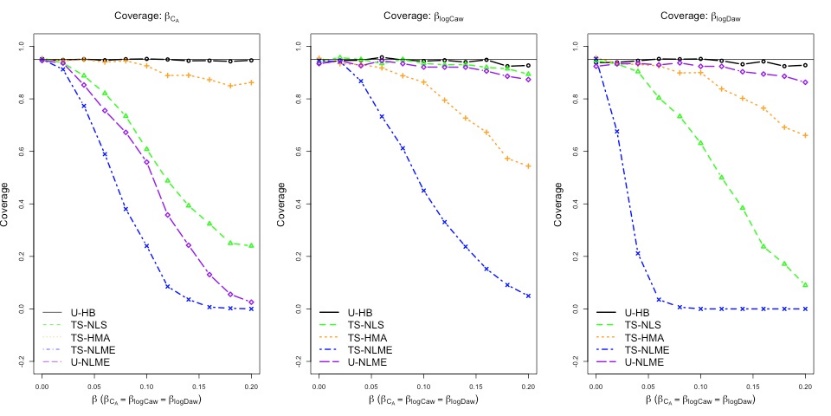

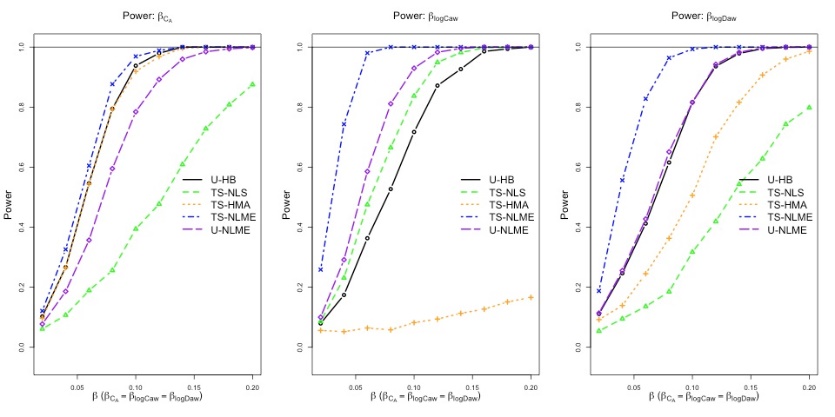


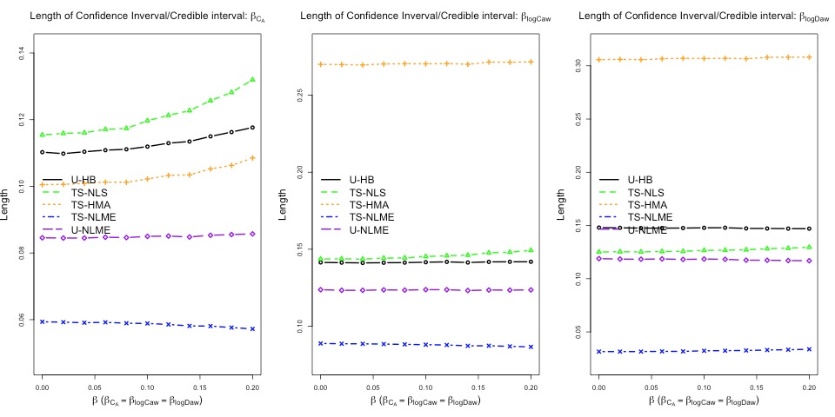


We see that $\beta_{C_{A}}$ was underestimated by all models except U-HB, with the degree of negative bias increasing with effect size. U-HB and U-NLME showed least bias for $\beta_{{logC}_{aw}}$ and $\beta_{{logD}_{aw}}$ but in different directions. TS-NLS, TS-HMA and TS-NLME showed good power but it is because they have biased estimation, short confidence interval length, or low coverage. TS-NLME performed well for estimating $\beta_{{logC}_{aw}}$ but not for $\beta_{C_{A}}$ and $\beta_{{logD}_{aw}}$. U-NLME performed well for $\beta_{{logC}_{aw}}$ and $\beta_{{logD}_{aw}}$ but not for $\beta_{C_{A}}$.

**Supplementary Figure 2.2.** Relative bias (a), coverage (b), and power (c) CI length (d) of the selected estimation methods from Scenario 2 of the simulation study ($\beta_{C_{A}}$varied, $\beta_{{logC}_{aw}}=0$, and $\beta_{{logD}_{aw}}=0$).


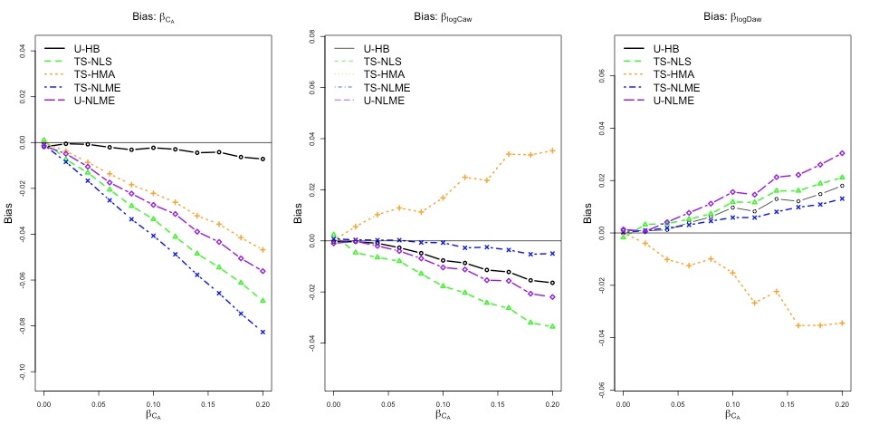


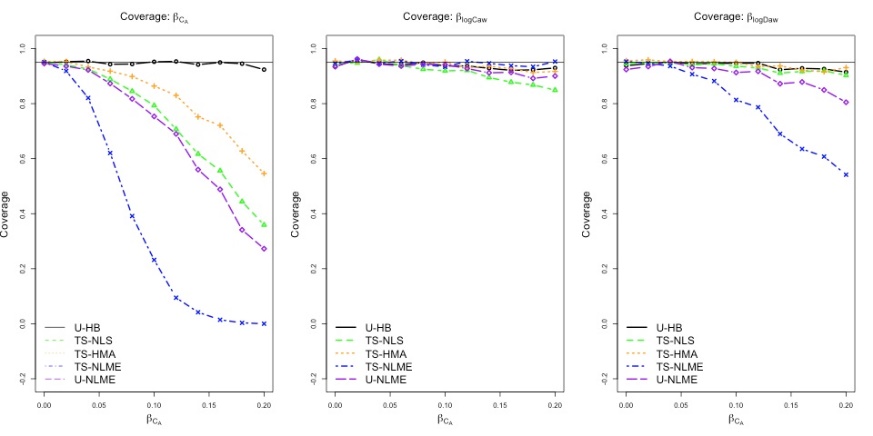


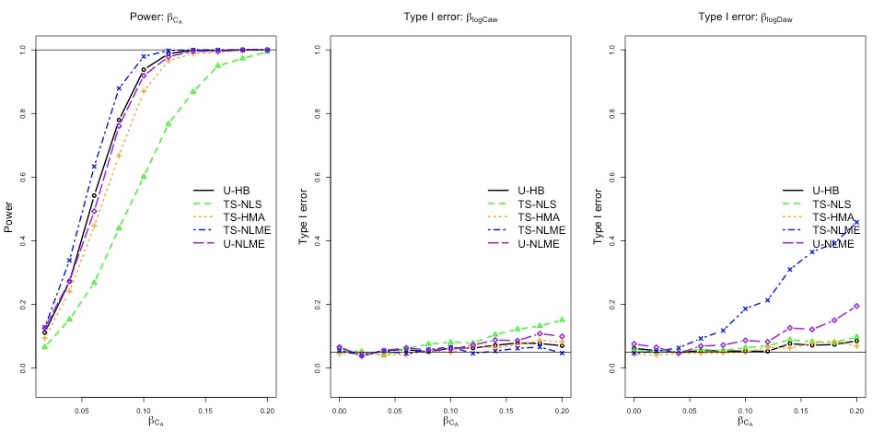


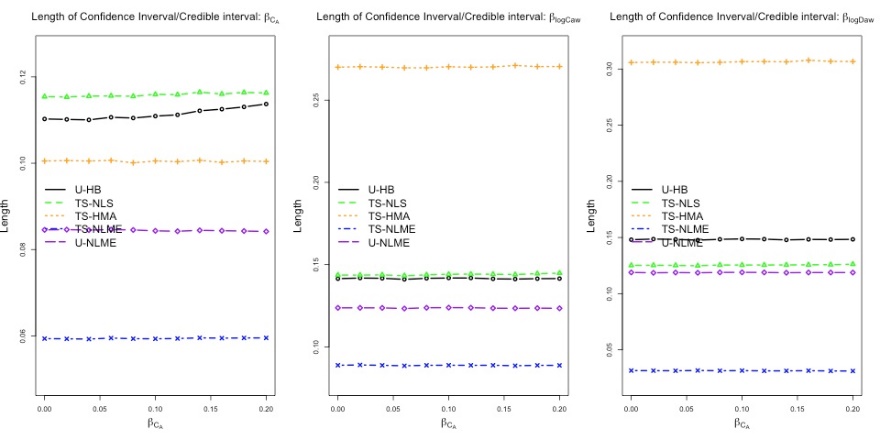


The directions of bias were changed in this scenario for $\beta_{{logC}_{aw}}$ and $\beta_{{logD}_{aw}}$. U-HB had good performance and was very stable, unlike U-NLME, which had high type I error due to its bias and narrow CI length.

**Supplementary Figure 2.3.** Relative bias (a), coverage (b), and power (c) CI length (d) of the selected estimation methods from Scenario 3 of the simulation study ($\beta_{C_{A}}=0$, $\beta_{{logC}_{aw}}$varied, and $\beta_{{logD}_{aw}}=0$).


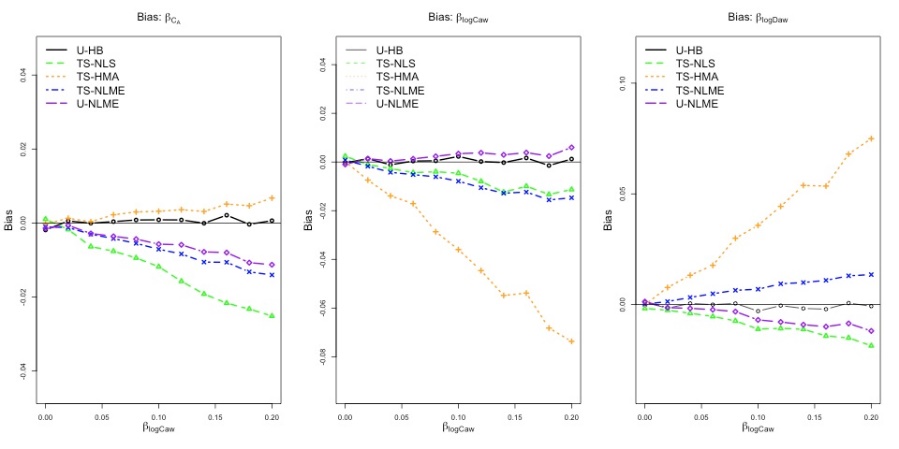

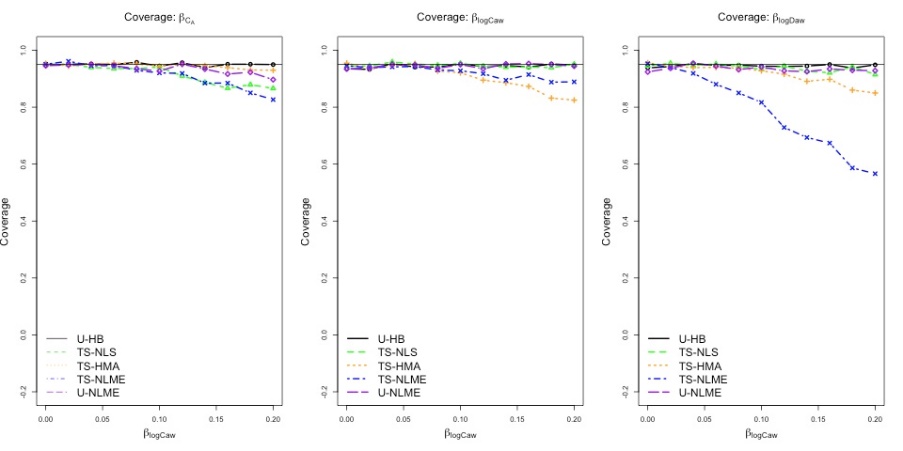

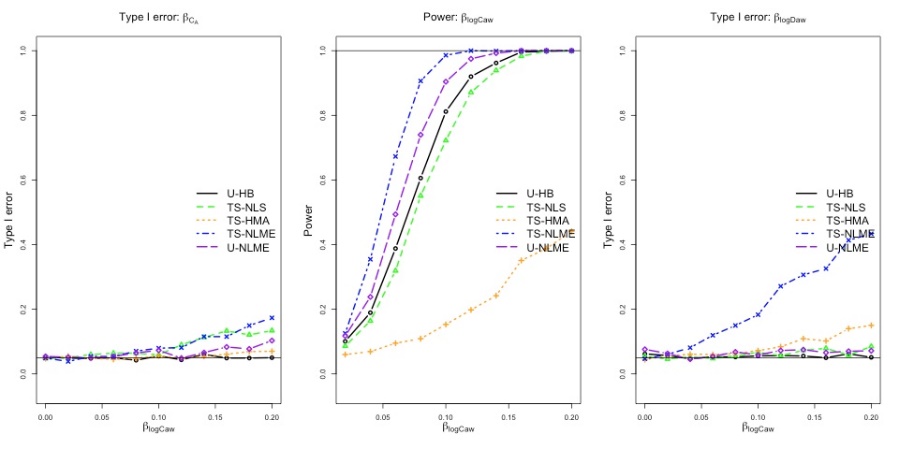


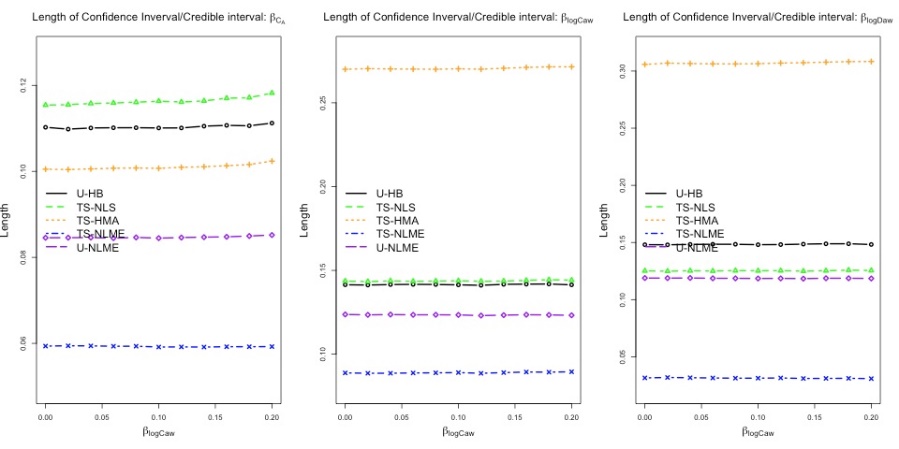


The direction of bias flipped again for $\beta_{{logC}_{aw}}$ and $\beta_{{logD}_{aw}}$. U-HB was still the best and most stable model.

**Supplementary Figure 2.4.** Relative bias (a), coverage (b), and power (c) CI length (d) of the selected estimation methods from Scenario 4 of the simulation study ($\beta_{C_{A}}=0$, $\beta_{{logC}_{aw}}=0$, and $\beta_{{logD}_{aw}}$varied).


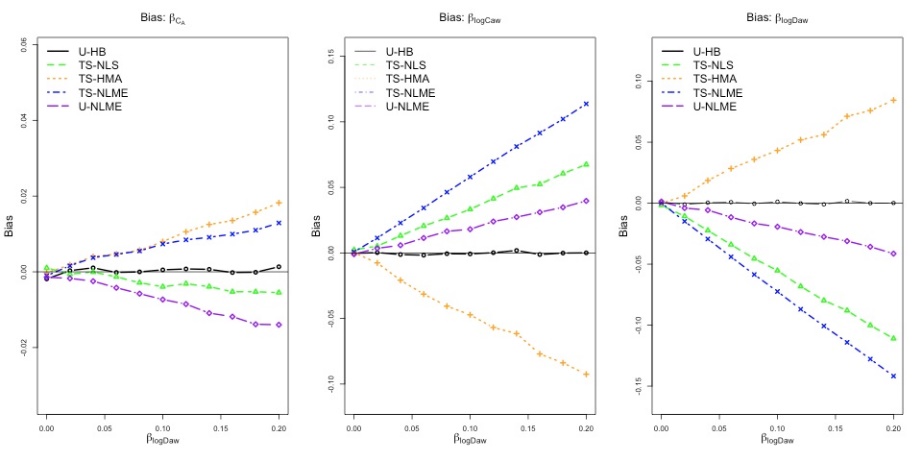

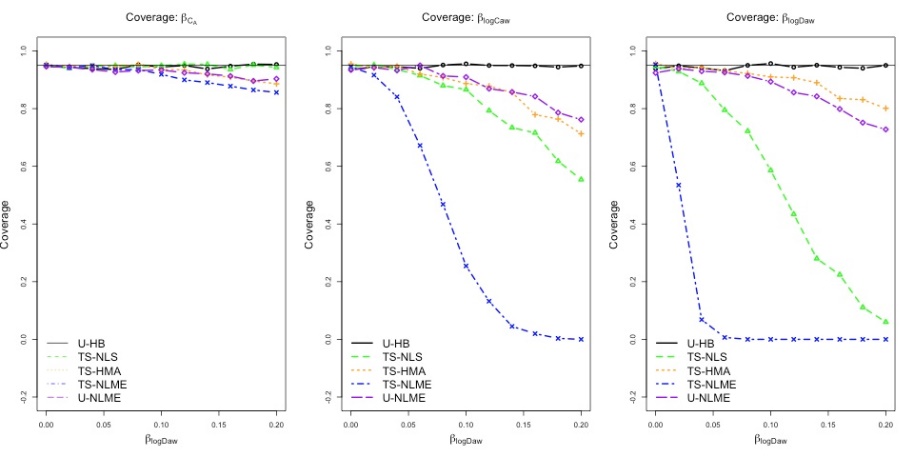

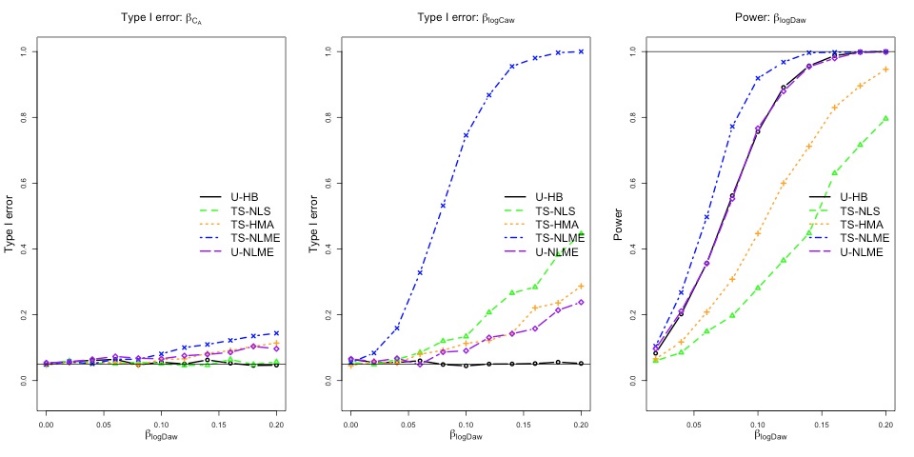


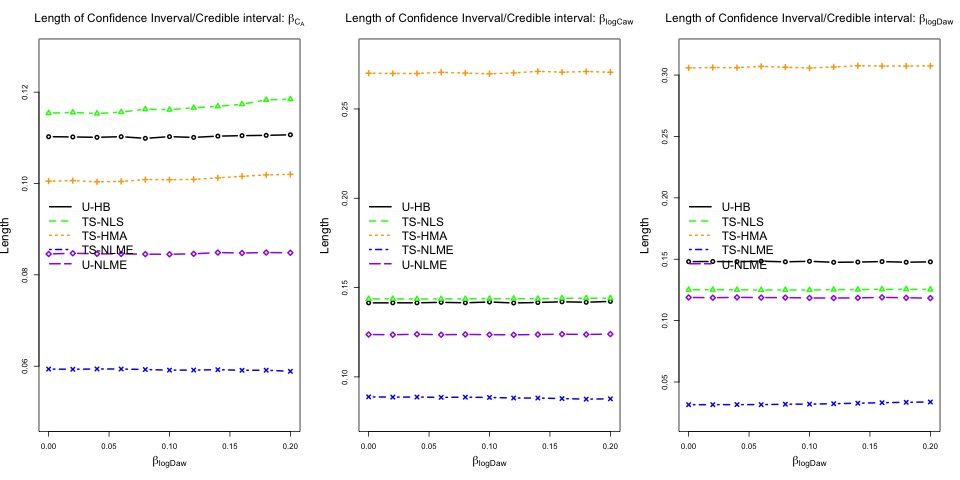


Estimation of $\beta_{{logC}_{aw}}$ was affected greatly by the increasing size of $\beta_{{logD}_{aw}}$for all models except U-HB. While estimation of $\beta_{C_{A}}$ was hardly affected.

**Supplementary Figure 2.5.** Relative bias (a), coverage (b), and power (c) CI length (d) of the selected estimation methods from Scenario 5 of the simulation study ($\beta_{C_{A}}=0$, $\beta_{{logC}_{aw}}=\beta_{{logD}_{aw}}$ varied).


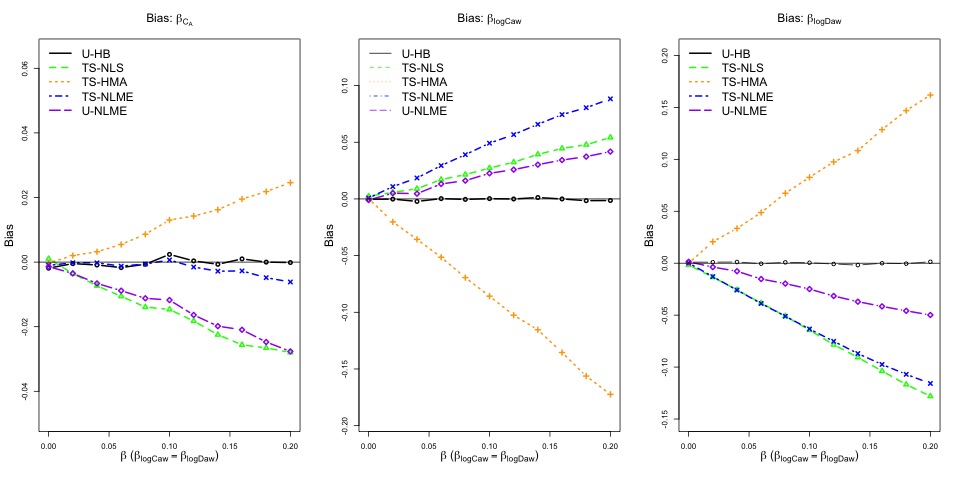

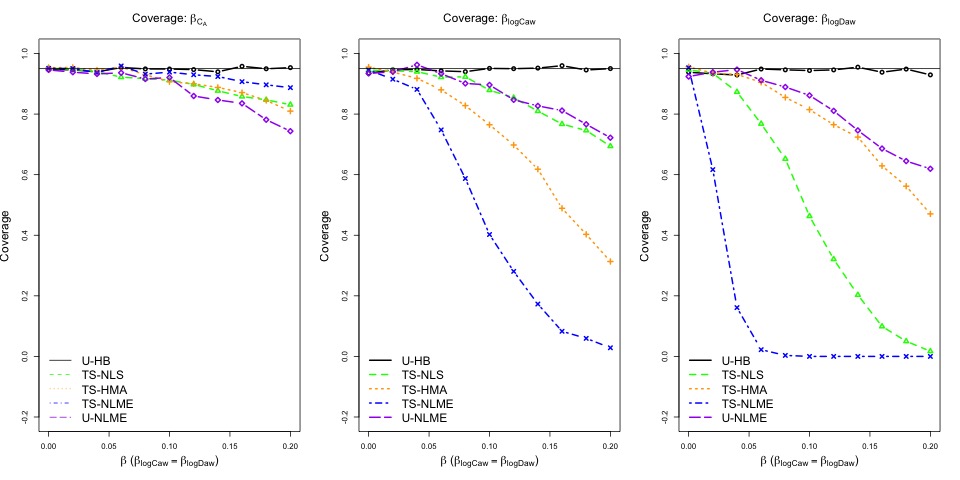

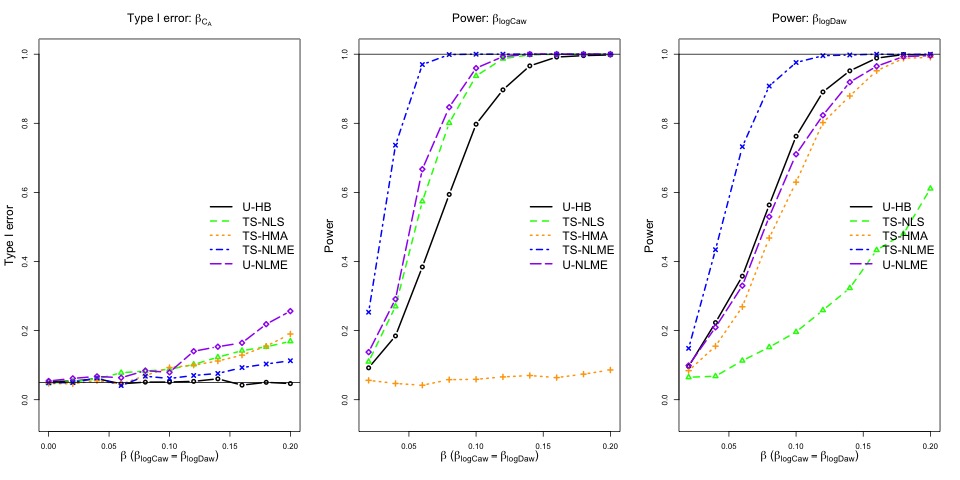


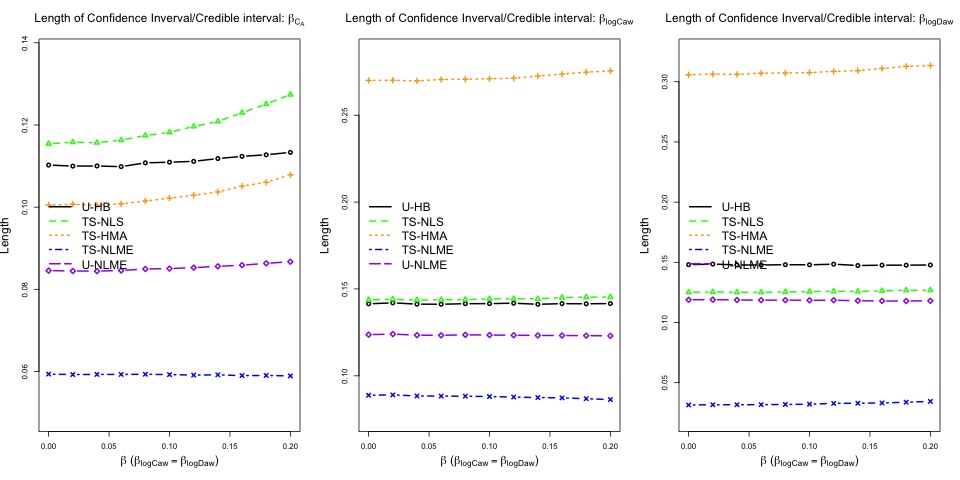


Overall, the U-HB model again had the best performance.

**Supplementary Figure 2.6.** Relative bias (a), coverage (b), and power (c) CI length (d) of the selected estimation methods from Scenario 6 of the simulation study ($\beta_{{logC}_{aw}}=0$ while $\beta_{C_{A}}=\beta_{{logD}_{aw}}$ varied).


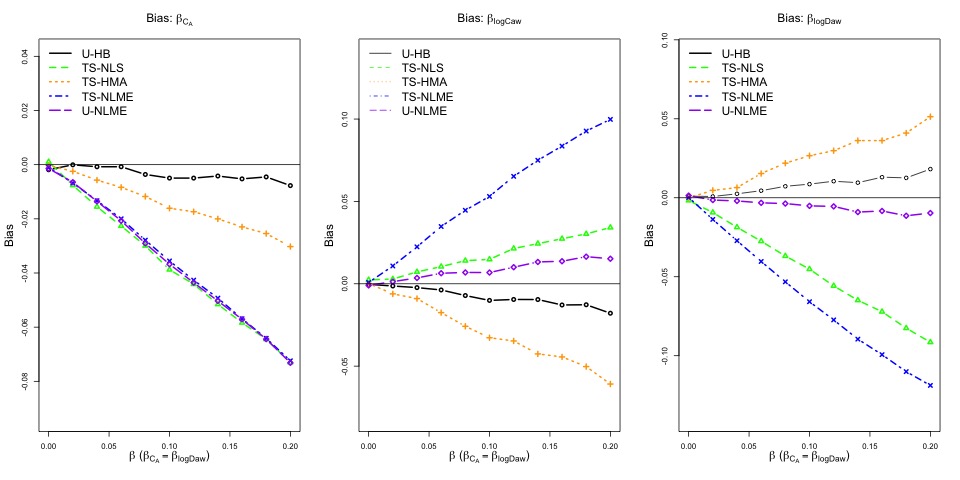

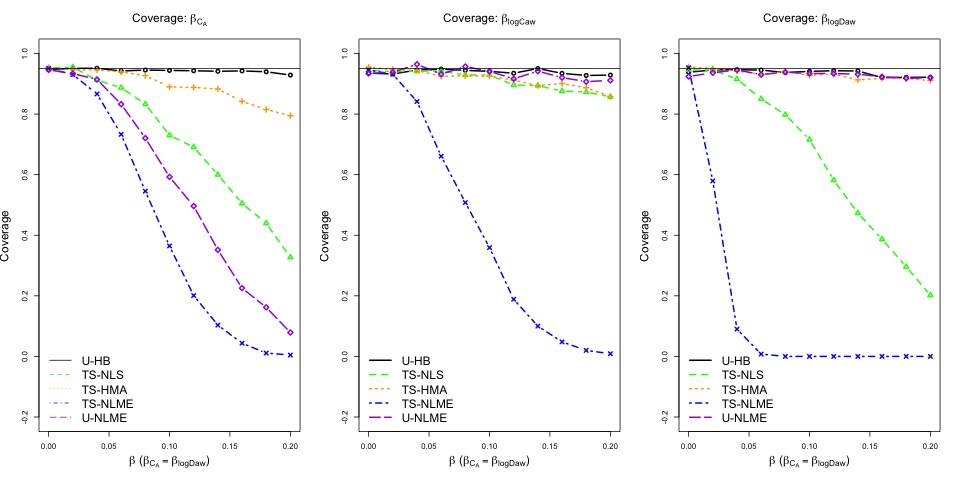

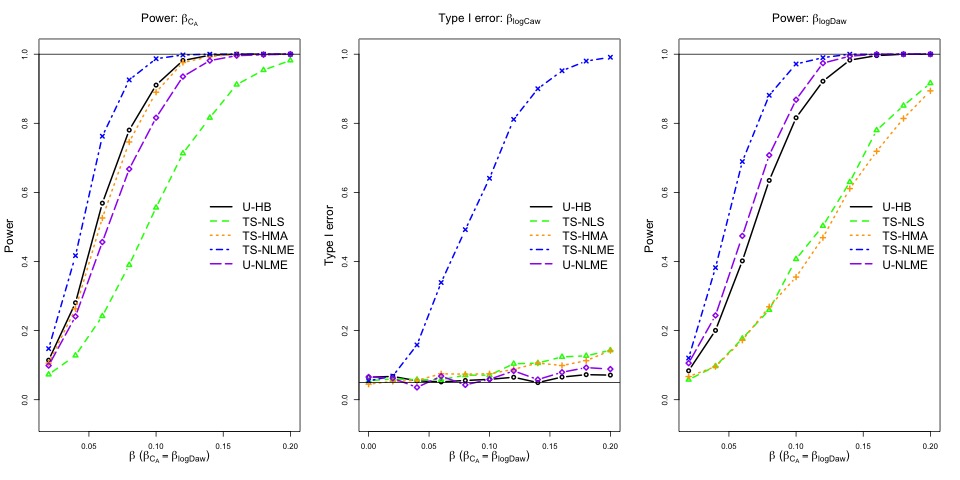


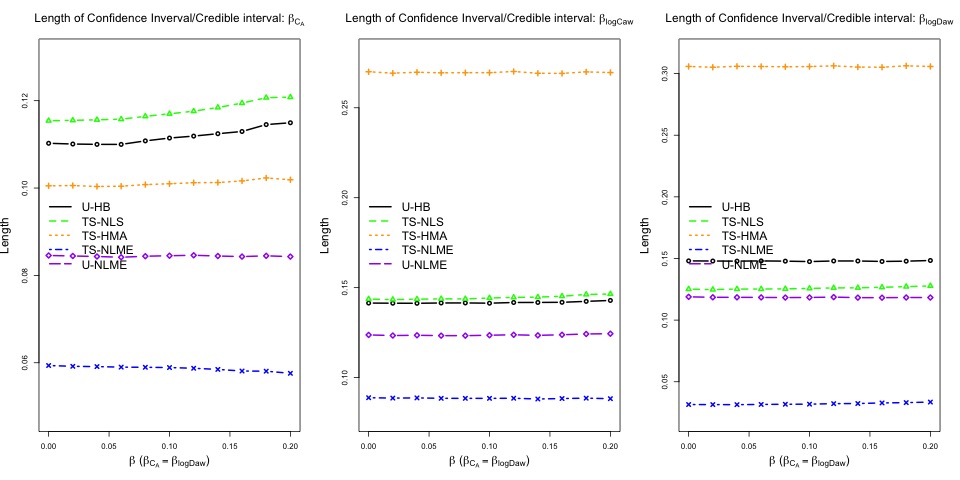


Overall, the U-HB model again had the best performance.

**Supplementary Figure 2.7.** Relative bias (a), coverage (b), and power (c) CI length (d) of the selected estimation methods from Scenario 7 of the simulation study ($\beta_{{logD}_{aw}}=0$ while $\beta_{C_{A}}{=\beta}_{{logC}_{aw}}$ varied).


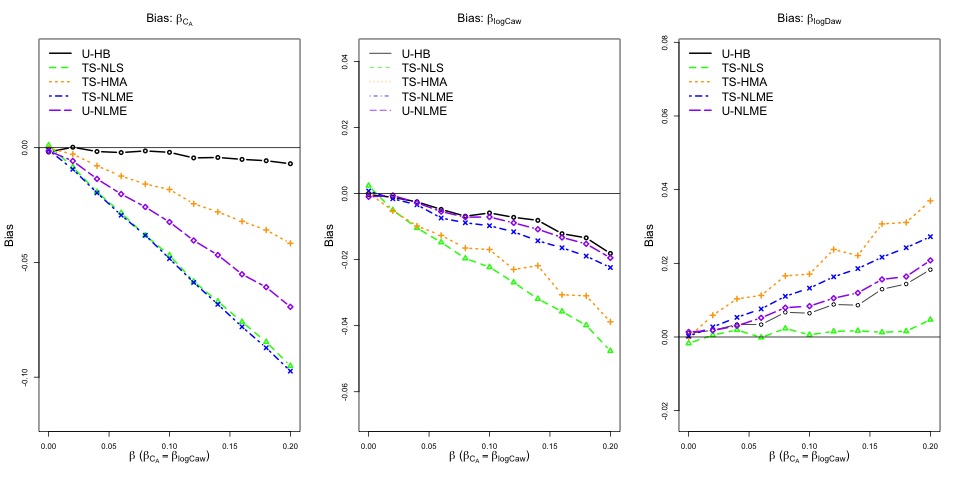

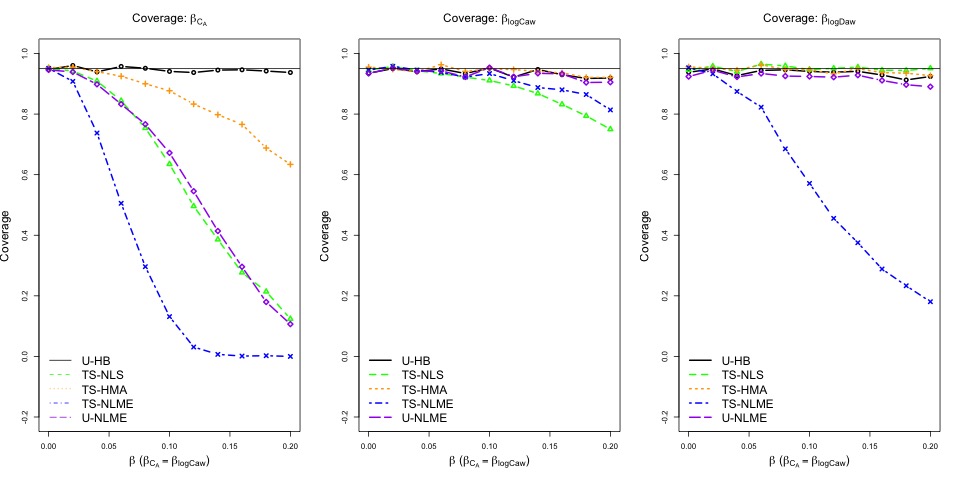

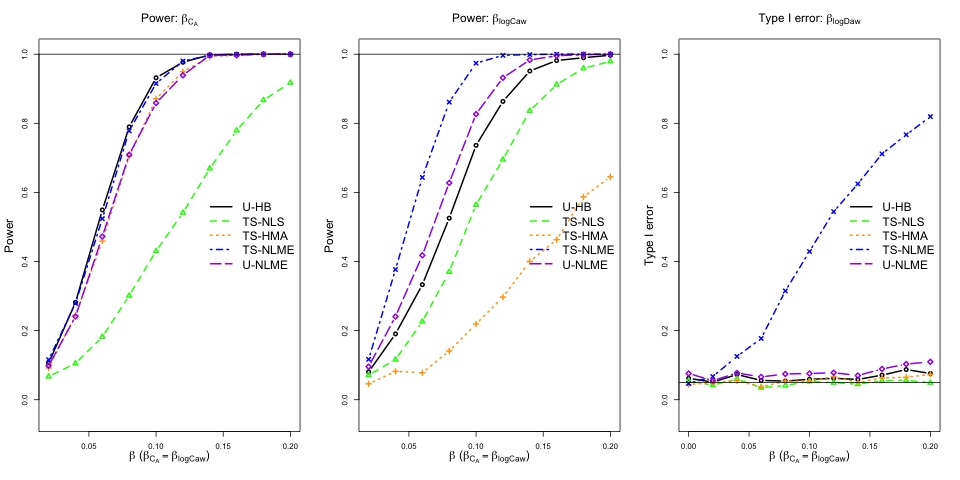


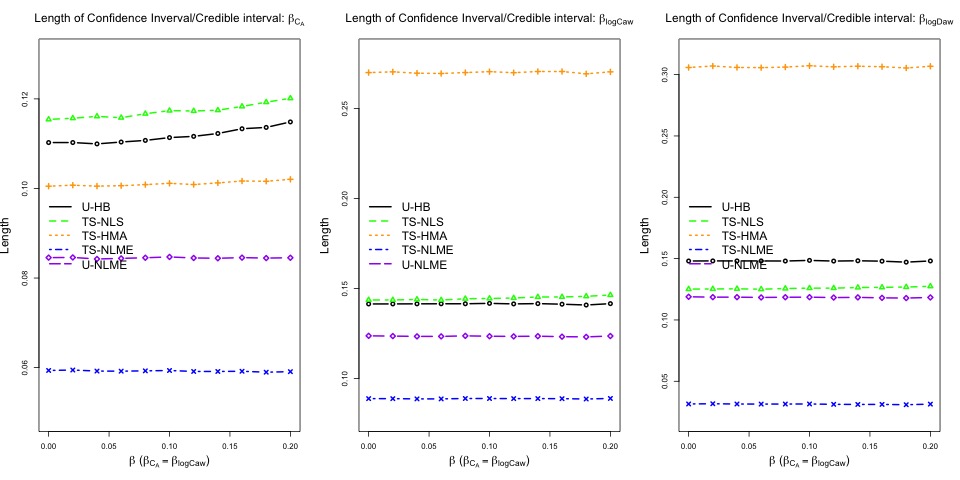


Overall, the U-HB model again had the best performance.

# **Section 3: Bias, Coverage, Power and Confidence Interval Length figures for** $\boldsymbol{\alpha}$**, by scenario**

**Supplementary Figure 3.1.** Relative bias (a), coverage (b), and power (c) CI length (d) of the selected estimation methods from Scenario 1 of the simulation study ($\beta_{C_{A}}$= $\beta_{{logC}_{aw}}$= $\beta_{{logD}_{aw}}$).


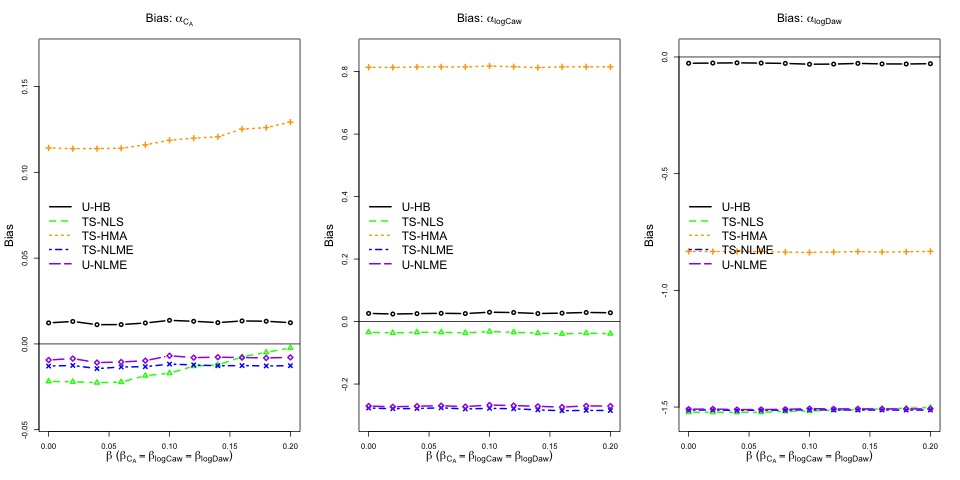

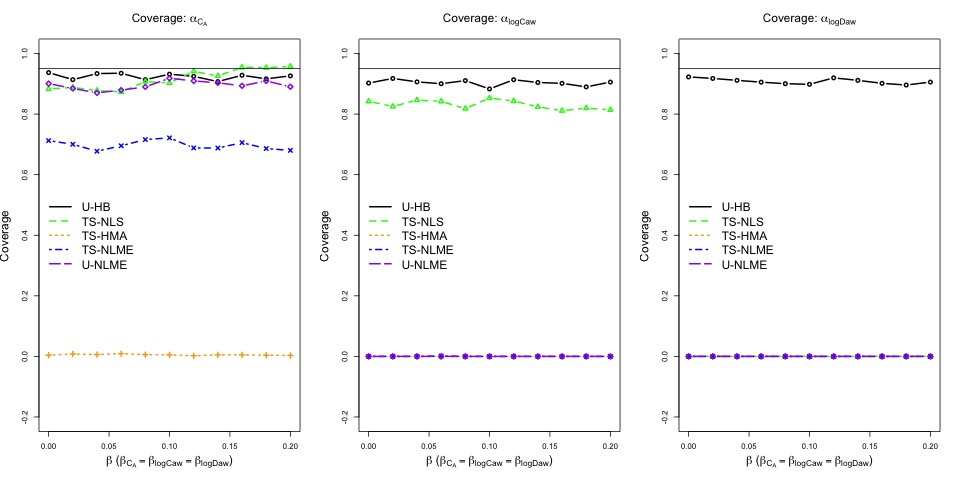

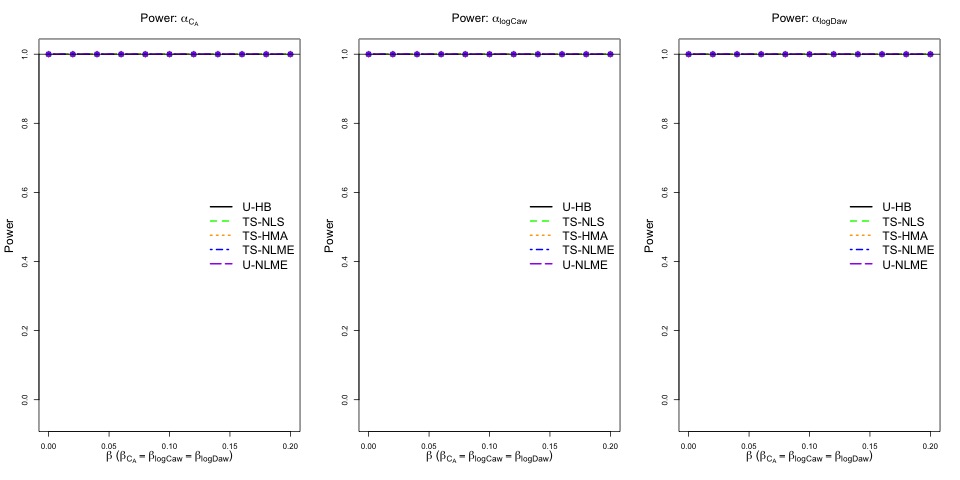


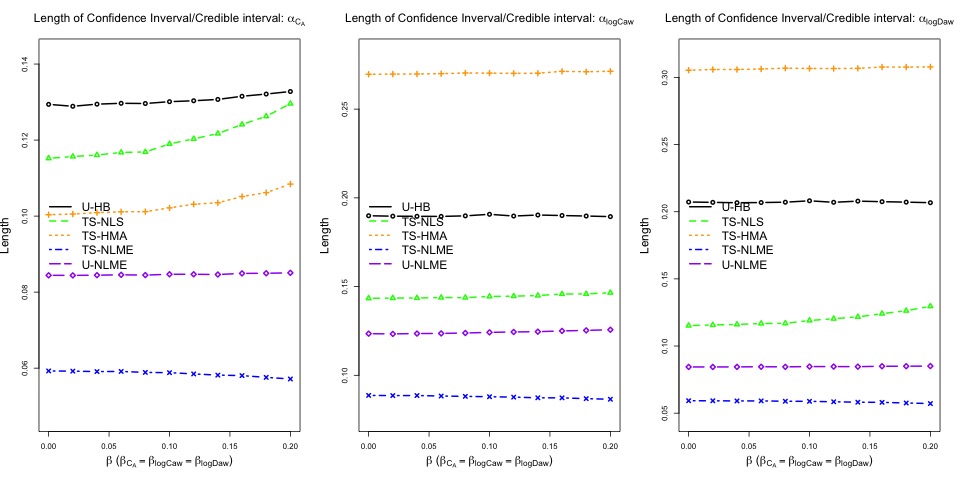


**Supplementary Figure 3.2.** Relative bias (a), coverage (b), and power (c) CI length (d) of the selected estimation methods from Scenario 2 of the simulation study ($\beta_{C_{A}}$varied, $\beta_{{logC}_{aw}}=0$, and $\beta_{{logD}_{aw}}=0$).


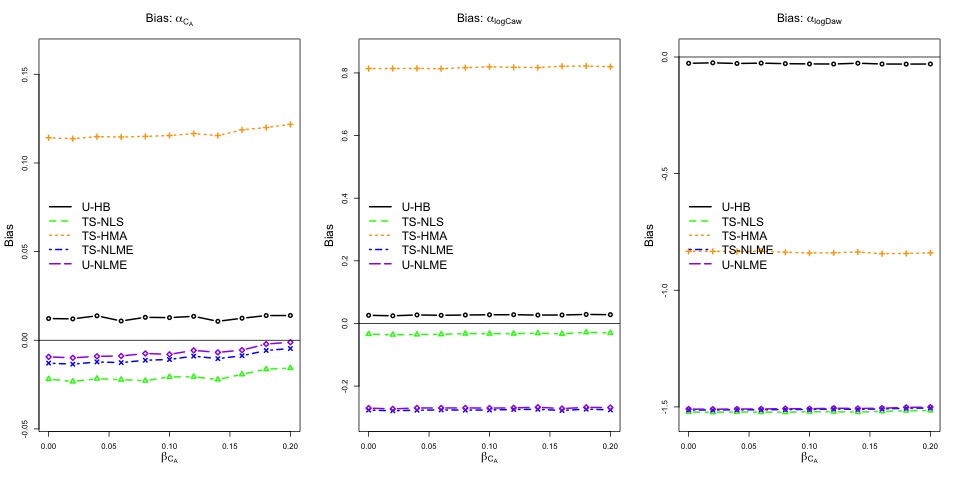

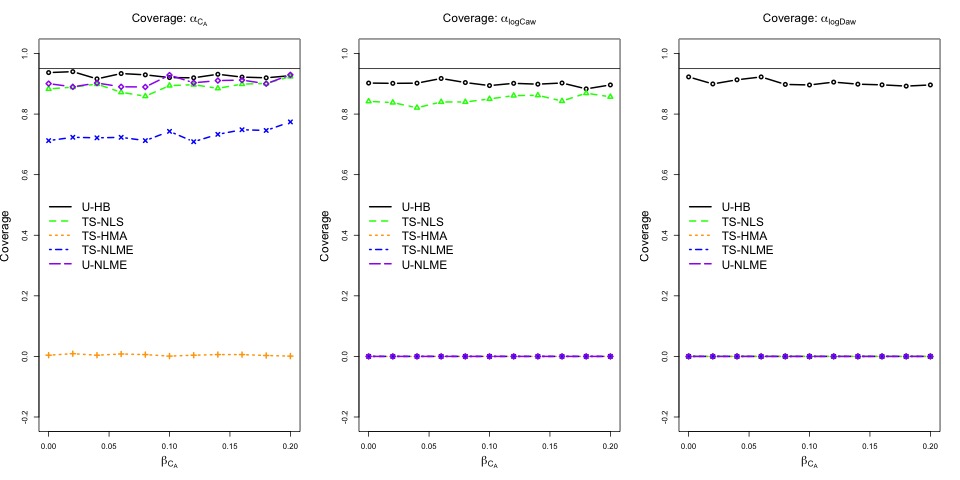

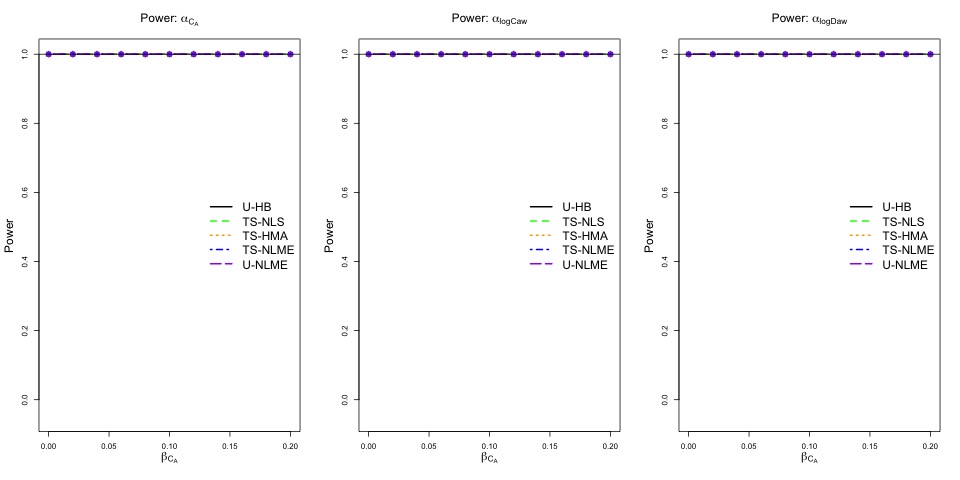


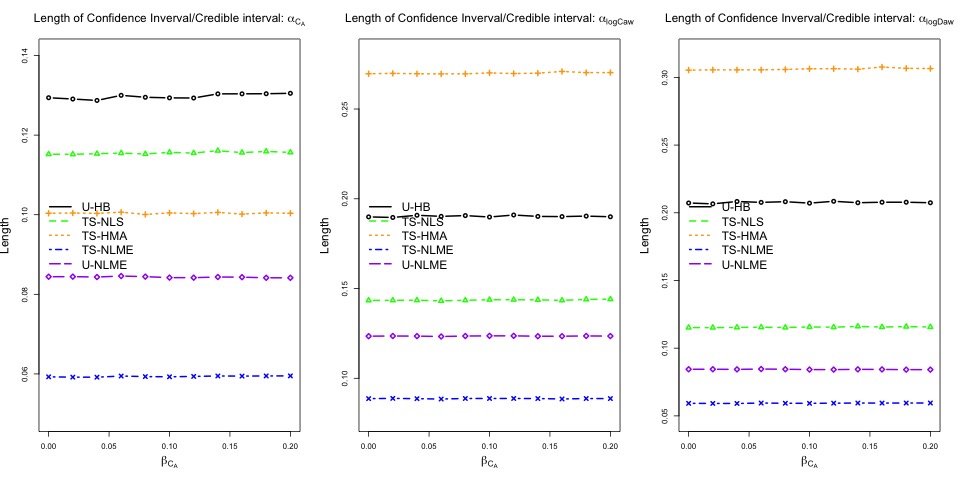


**Supplementary Figure 3.3.** Relative bias (a), coverage (b), and power (c) CI length (d) of the selected estimation methods from Scenario 3 of the simulation study ($\beta_{C_{A}}=0$, $\beta_{{logC}_{aw}}$varied, and $\beta_{{logD}_{aw}}=0$).


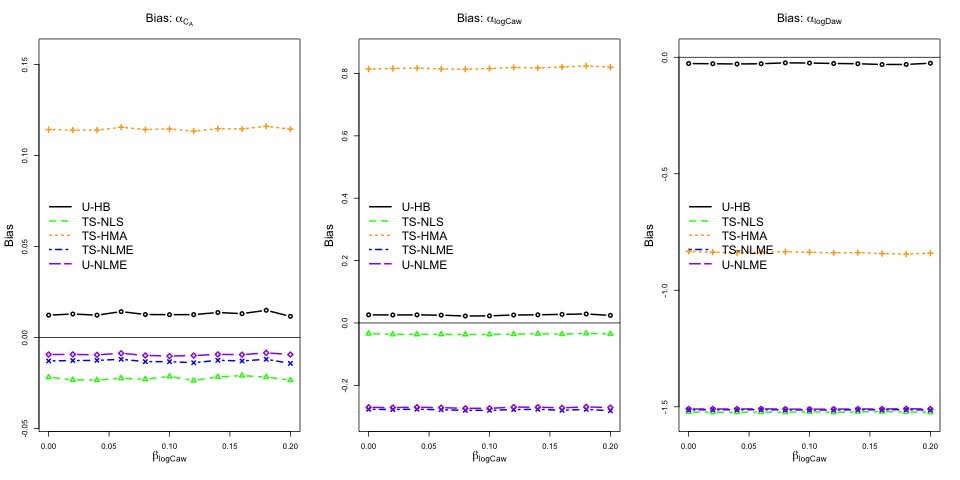

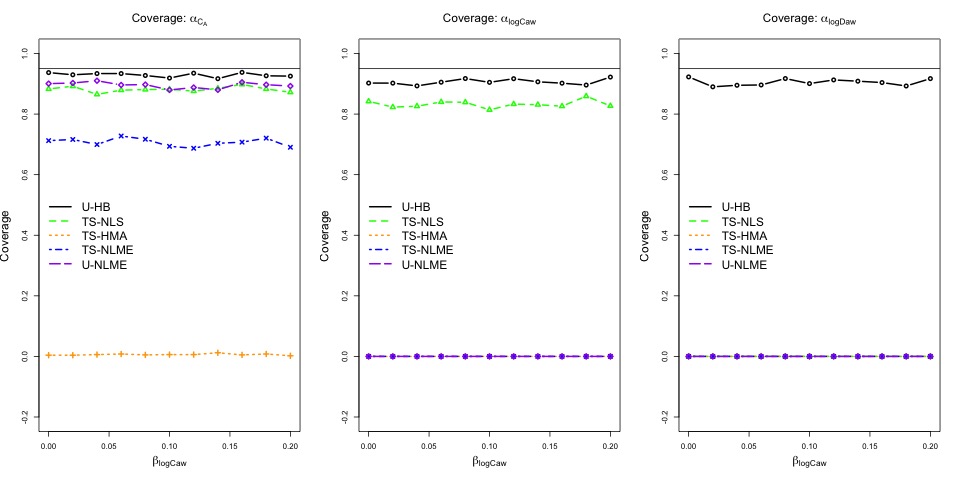

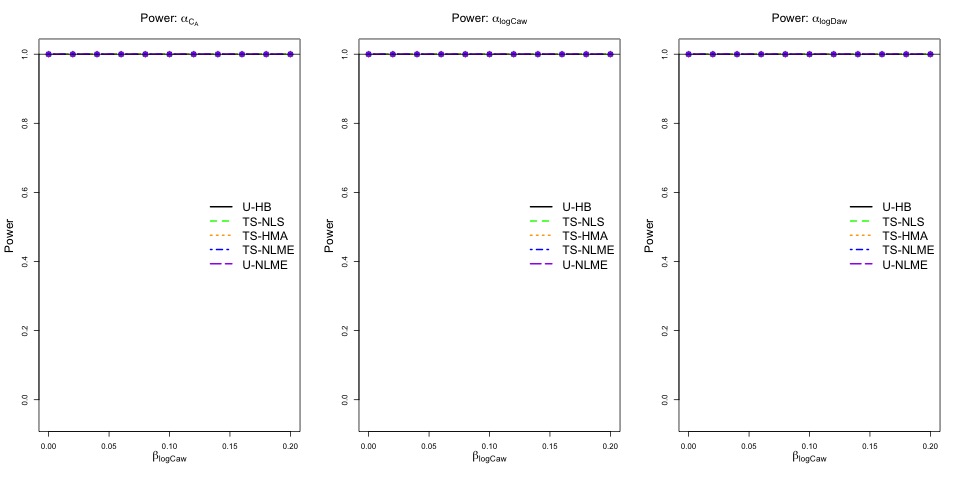


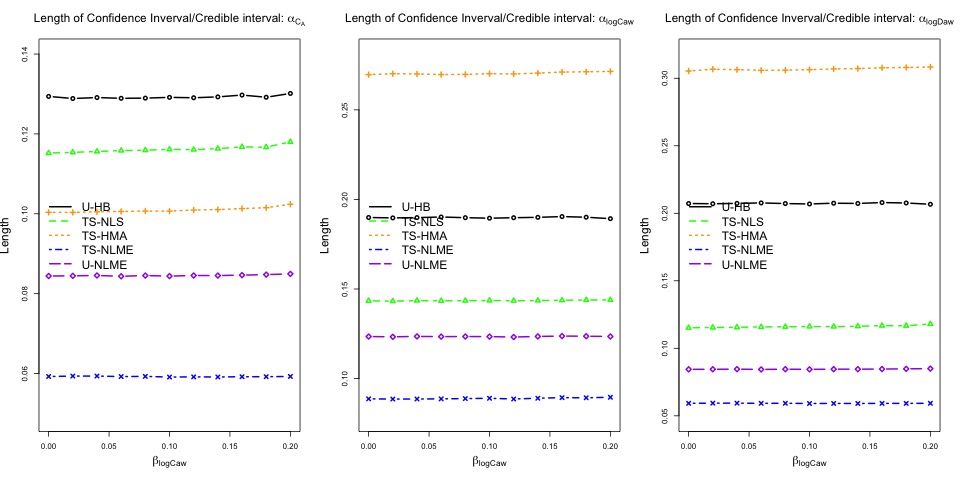


**Supplementary Figure 3.4.** Relative bias (a), coverage (b), and power (c) CI length (d) of the selected estimation methods from Scenario 4 of the simulation study ($\beta_{C_{A}}=0$, $\beta_{{logC}_{aw}}=0$, and $\beta_{{logD}_{aw}}$varied).


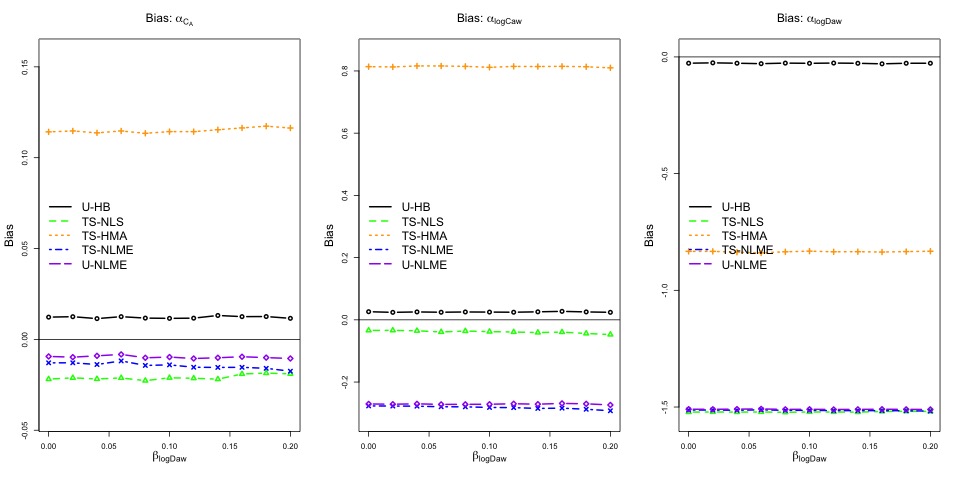

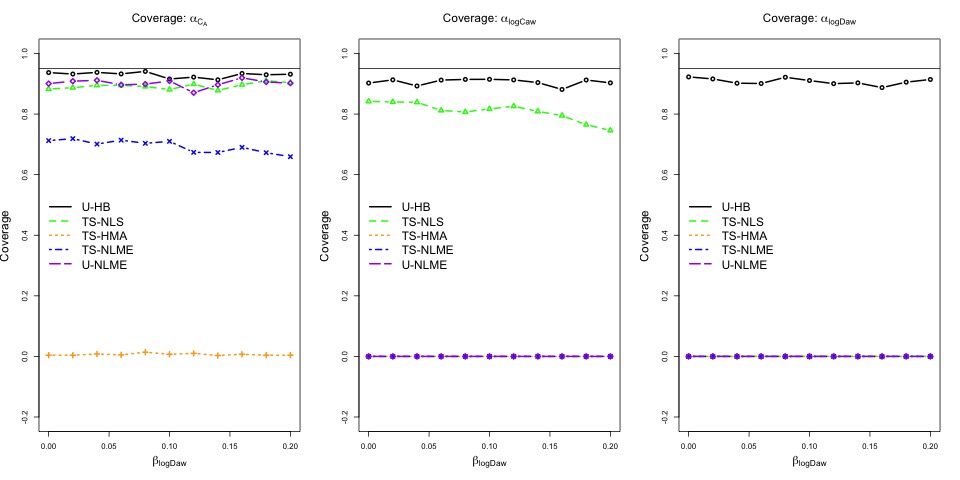

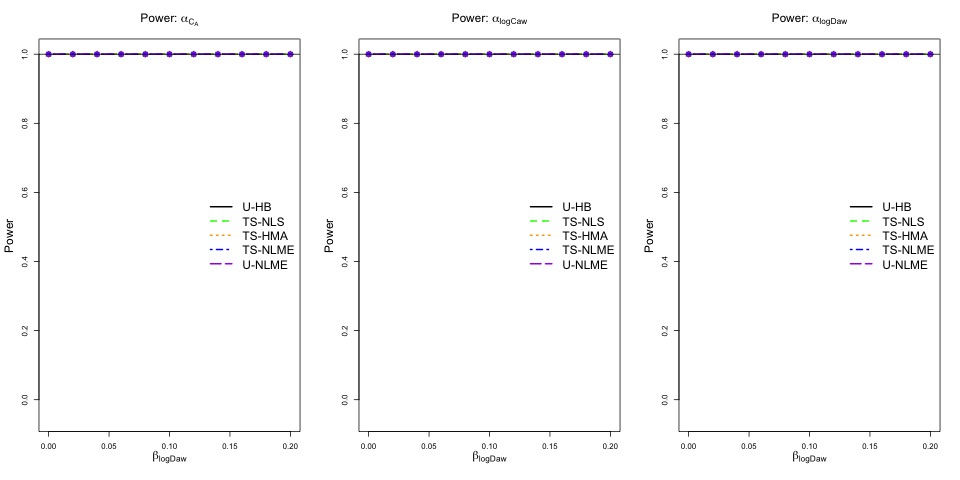


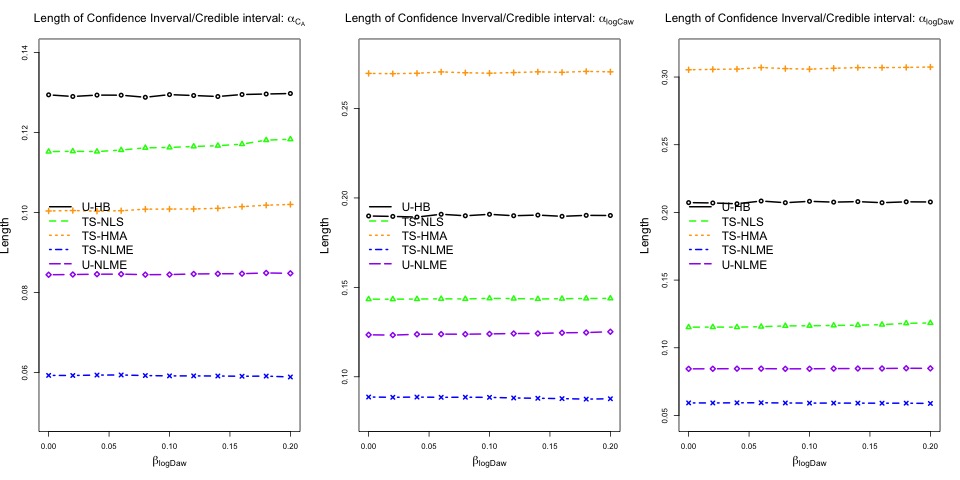


**Supplementary Figure 3.5.** Relative bias (a), coverage (b), and power (c) CI length (d) of the selected estimation methods from Scenario 5 of the simulation study ($\beta_{C_{A}}=0$, $\beta_{{logC}_{aw}}=\beta_{{logD}_{aw}}$ varied).
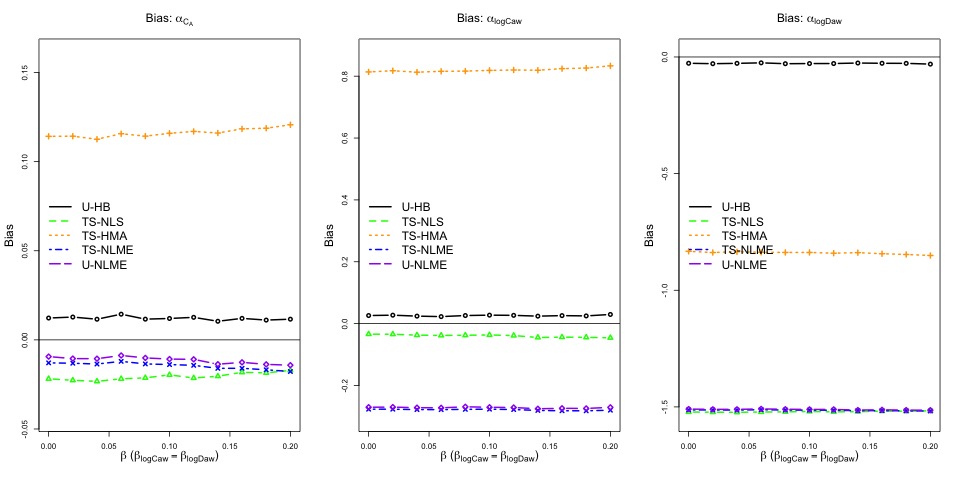

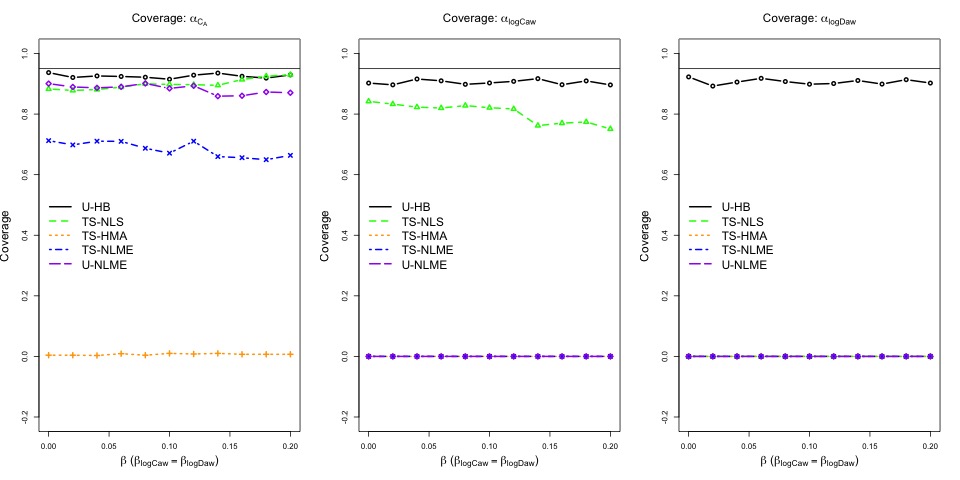

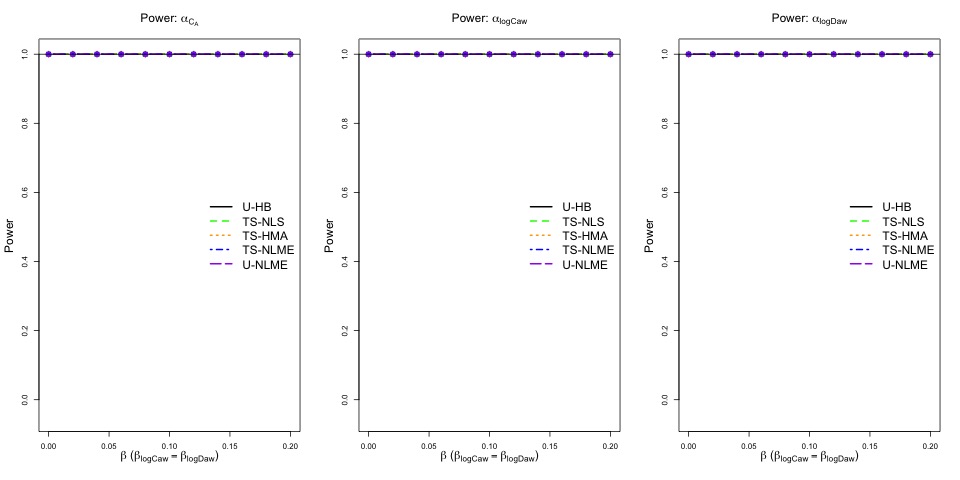


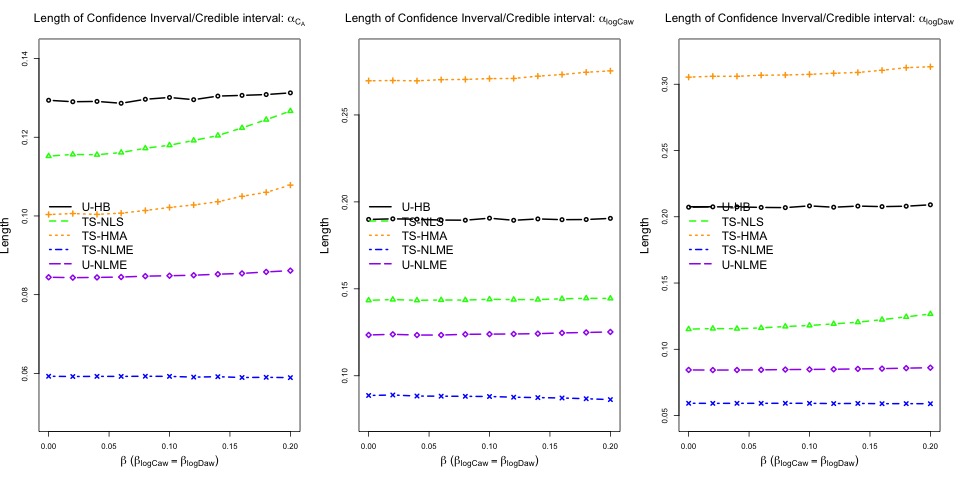


**Supplementary Figure 3.6.** Relative bias (a), coverage (b), and power (c) CI length (d) of the selected estimation methods from Scenario 6 of the simulation study ($\beta_{{logC}_{aw}}=0$ while $\beta_{C_{A}}=\beta_{{logD}_{aw}}$ varied).
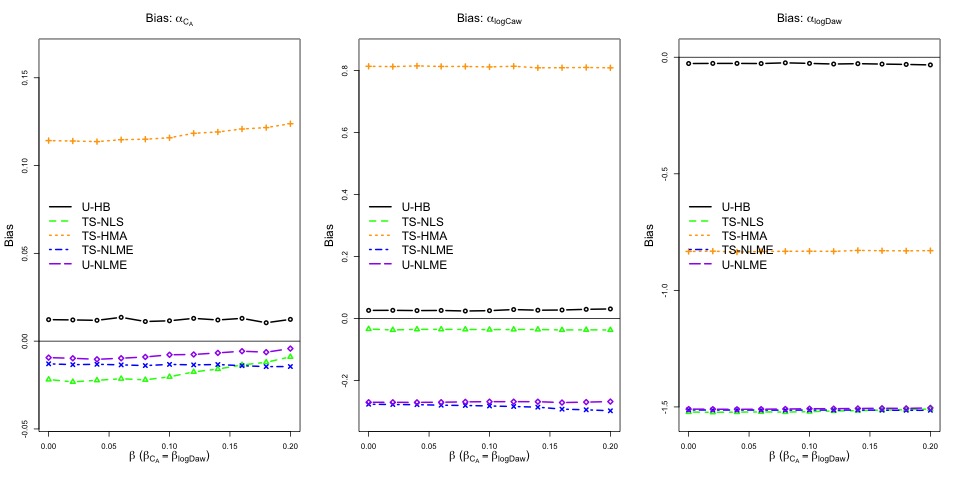

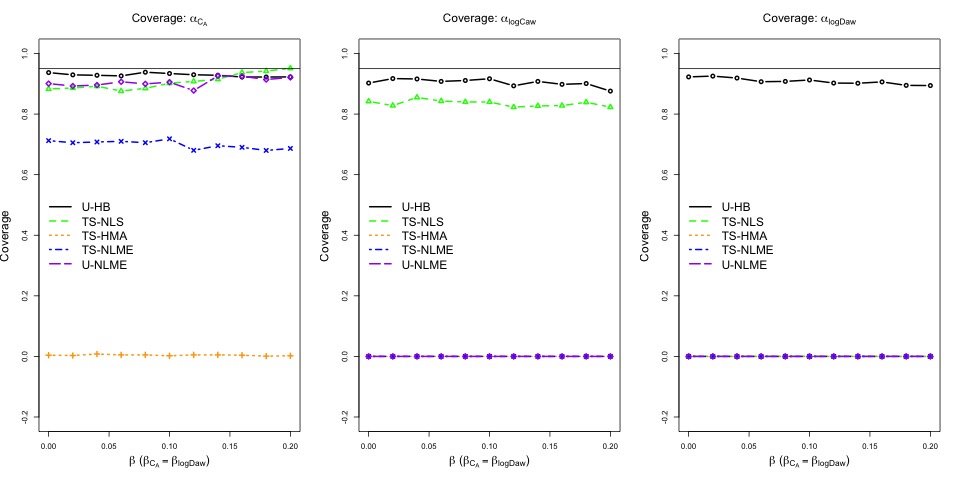

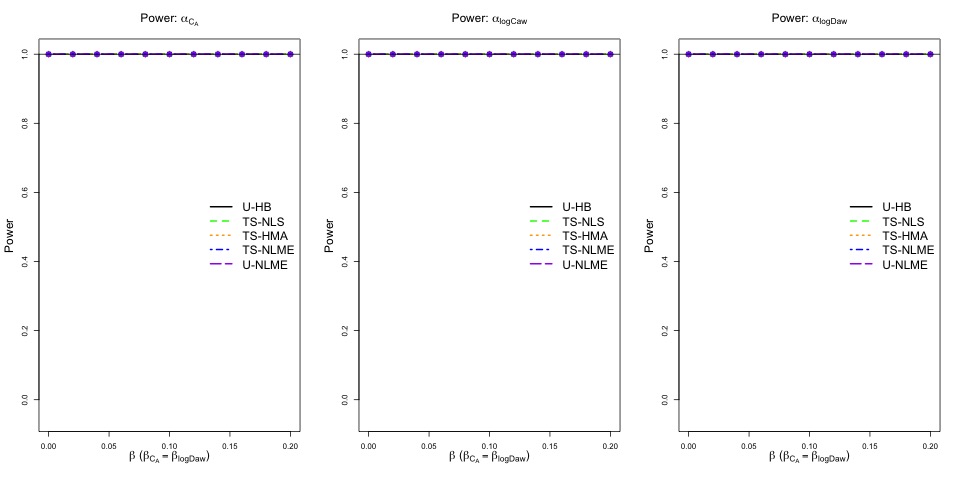


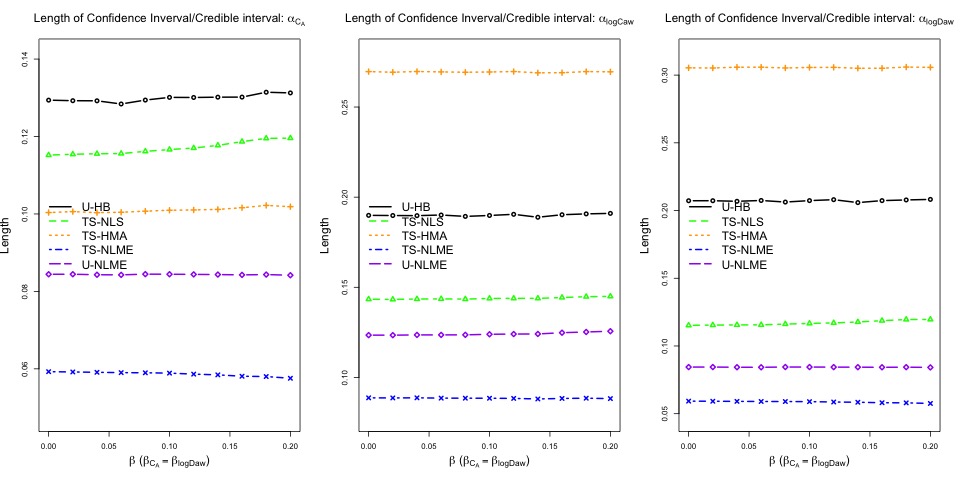


**Supplementary Figure 3.7.** Relative bias (a), coverage (b), and power (c) CI length (d) of the selected estimation methods from Scenario 7 of the simulation study ($\beta_{{logD}_{aw}}=0$ while $\beta_{C_{A}}{=\beta}_{{logC}_{aw}}$ varied).
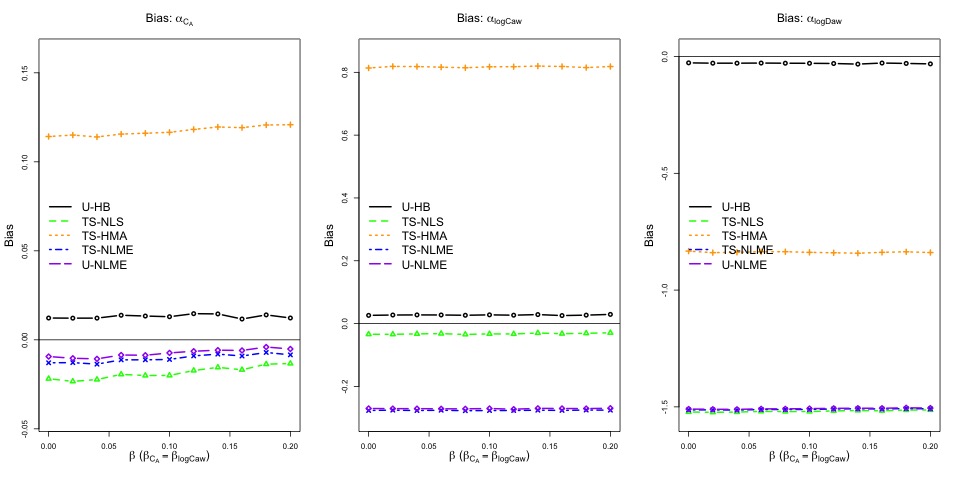

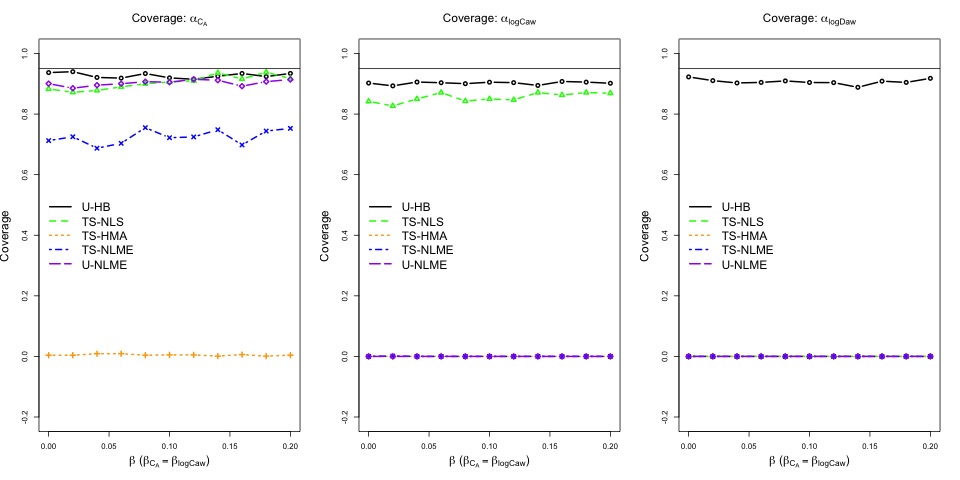

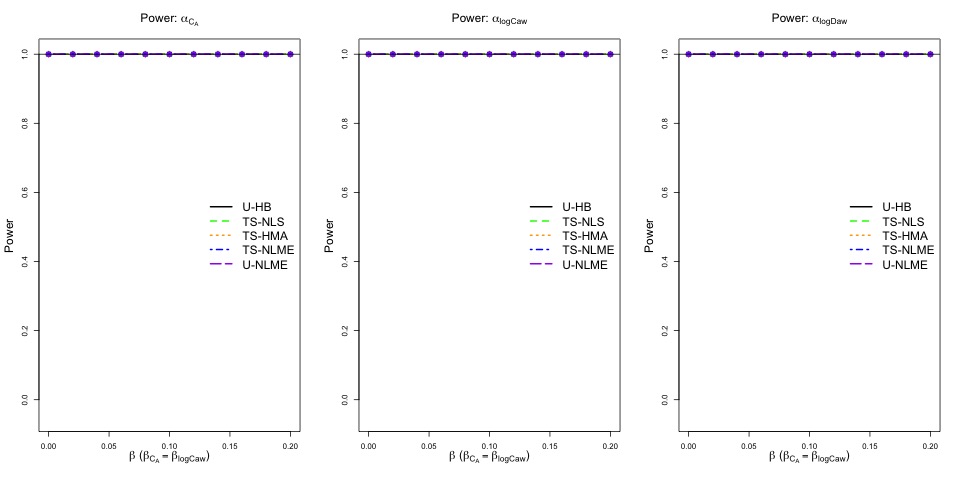


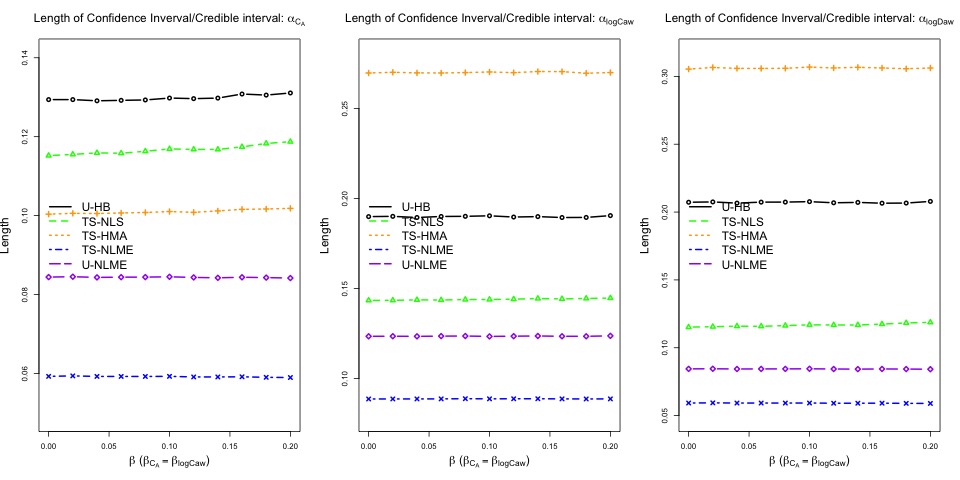


# **Section 4. Additional details on CHS results**

For CHS participants, the estimated participant-level NO parameters were highly correlated (Pearson’s R>0.92) across U-HB, U-NLME and TS-NLME methods under the *C_aw_* parameterization (Supplementary Figure 4.1) and also under the *J’_aw_* parameterization (Supplementary Figure 4.2). As shown in Supplementary Figure 4.3, calculated *logJ_aw_* estimates from U-HB (*C_aw_* parameterization) were extremely highly correlated (Pearson’s R>0.999) with direct *logJ_aw_* estimates from U-HB (*J_aw_*-parameterization).

**Supplementary Figure 4.1**. Correlation of estimated participant-level NO parameters in the CHS across estimation methods using *C_aw_* parameterization: (left) *C_A_*, (middle) *logC_aw_*, (right) *logD_aw_*.


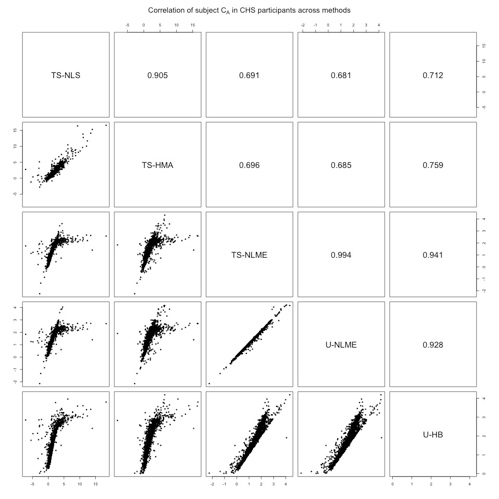

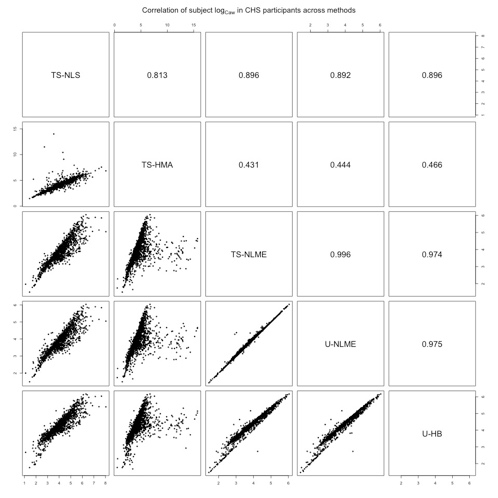

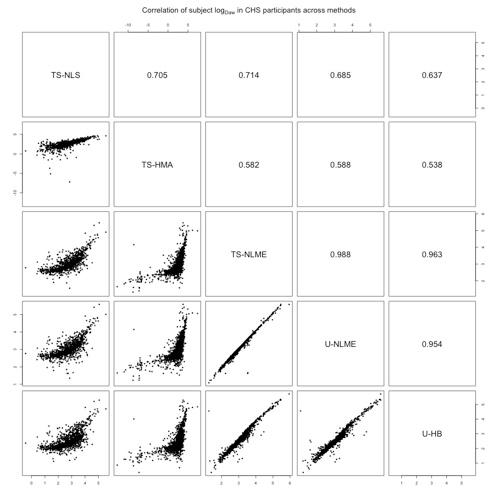


**Supplementary Figure 4.2**. Correlation of estimated participant-level NO parameters in the CHS across estimation methods using *J’_aw_* parameterization: (left) *C_A_*, (middle) *logJ’_aw_*, (right) *logD_aw_*.


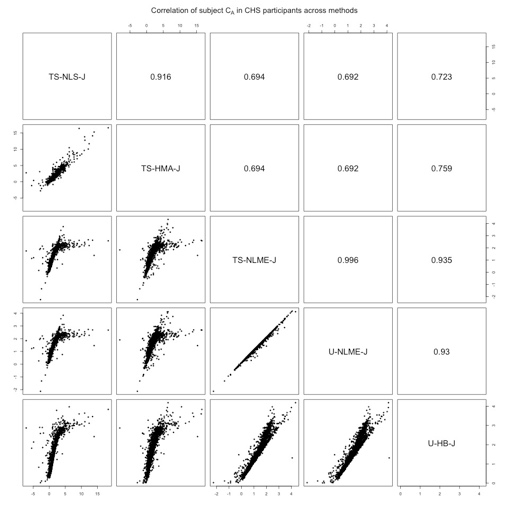

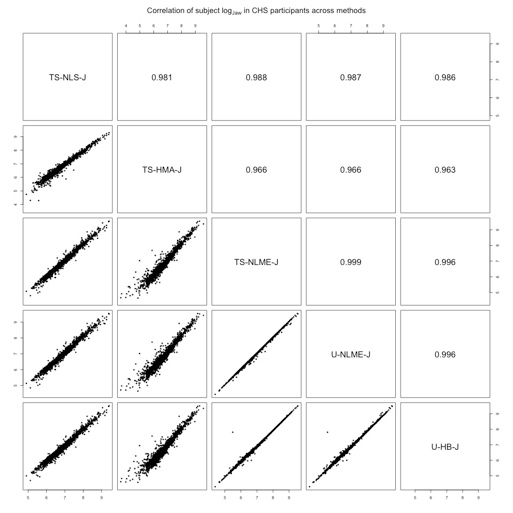

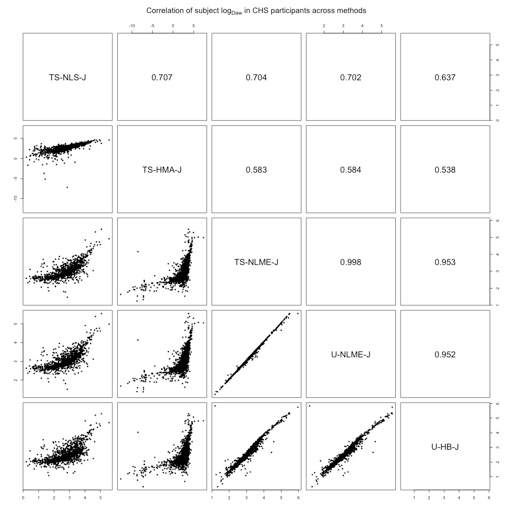


**Supplementary Figure 4.3.** Comparison of estimated participant-level *logJ’_aw_* in the CHS from *J’_aw_* and *C_aw_* parameterizations of U-HB.


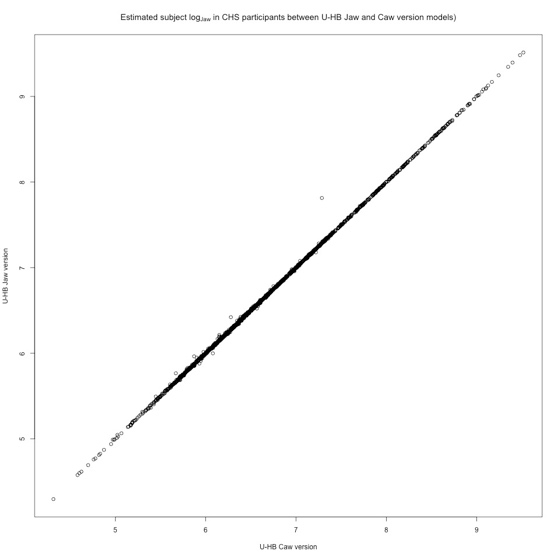


**Supplementary Figure 4.4**. Analysis of CHS data: estimated population-level mean NO parameters when traffic-related air pollution was 0 ($\hat{\alpha}$ and 95% CI) using the selected methods,^*^ with no adjustments for covariates.


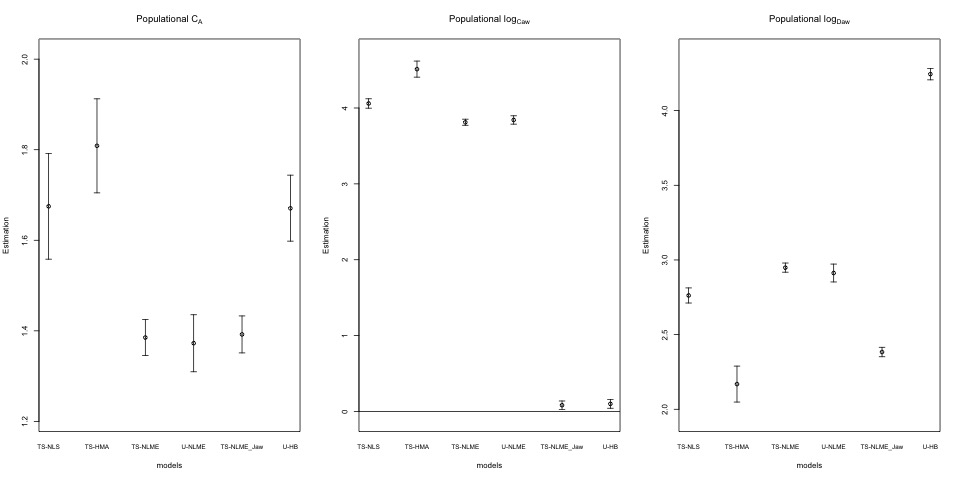


^*^ TS-NLME(*J’_aw_*) is the previously published model using a *J’_aw_* parameterization of TS-NLME.

**Supplementary Table 4.1**. Analysis of CHS data: estimated population-level parameters (and 95% CI) using the selected methods,^*^ in models that include traffic-related air pollution, age, sex, and asthma.

| **Estimated parameter (95% CI)** | **TS-NLS** | **TS-HMA** | **TS-NLME(*J’_aw_*)** | **TS-NLME** | **U-NLME** | **U-HB** |
| --- | --- | --- | --- | --- | --- | --- |
| $\alpha^{C_{A}}$ | 1.48 (1.32, 1.64) | 1.58 (1.44, 1.71) | 1.30 (1.24,1.35) | 1.29 (1.24,1.34) | 1.25 (1.17,1.33) | 1.51 (1.43,1.60) |
| $\beta_{\text{traffic}}^{C_{A}}$ | 0.23 (0.06, 0.40) | 0.32 (0.18, 0.46) | 0.08 (0.03,0.14) | 0.08 (0.03,0.14) | 0.13 (0.05,0.22) | 0.15 (0.66,0.24) |
| $\beta_{\text{age}}^{C_{A}}$ | 0.52 (0.28, 0.76) | 0.65 (0.43, 0.87) | -0.01 (-0.05,0.03) | -0.01 (-0.04,0.02) | -0.03 (-0.09,0.02) | -0.02 (-0.07,0.04) |
| $\beta_{\text{male}}^{C_{A}}$ | 0.17 (-0.03, 0.37) | 0.23 (0.05, 0.40) | 0.10 (0.03,0.17) | 0.10 (0.03,0.17) | 0.14 (0.03,0.25) | 0.17 (0.07,0.28) |
| $\beta_{\text{asthma}}^{C_{A}}$ | -0.001 (-0.10, 0.10) | -0.03 (-0.12, 0.06) | 0.24 (0.15,0.33) | 0.24 (0.15,0.32) | 0.29 (0.14,0.43) | 0.41 (0.26,0.55) |
| $\alpha^{\mathrm{logC}_{aw}}$ | 3.92 (3.84, 4.00) | 4.39 (4.25, 4.54) | 3.71 (3.65,3.76 | 3.68 (3.63,3.74) | 3.70 (3.62,3.77) | 4.13 (4.02,4.23) |
| $\beta_{\text{traffic}}^{\mathrm{logC}_{aw}}$ | 0.04 (-0.05, 0.13) | 0.16 (0.02, 0.31) | 0.02 (-0.04,0.07) | 0.01 (-0.04,0.07) | -0.03 (-0.09,0.06) | -0.02 (-0.12,0.07) |
| $\beta_{\text{age}}^{\mathrm{logC}_{aw}}$ | 0.46 (0.34, 0.59) | 0.42 (0.20, 0.65) | 0.05 (0.01,0.08) | 0.05 (0.02,0.08) | 0.06 (0.01,0.11) | 0.07 (0.01,0.13) |
| $\beta_{\text{male}}^{\mathrm{logC}_{aw}}$ | 0.09 (-0.01, 0.20) | 0.08 (-0.11, 0.26) | 0.12 (0.05,0.19) | 0.13 (0.05,0.19) | 0.14 (0.04,0.23) | 0.13 (0.02,0.25) |
| $\beta_{\text{asthma}}^{\mathrm{logC}_{aw}}$ | 0.07 (0.02, 0.13) | 0.02 (-0.07, 0.11) | 0.36 (0.28,0.44) | 0.37 (0.28,0.45) | 0.38 (0.27,0.50) | 0.33 (0.19,0.48) |
| $\alpha^{\mathrm{logD}_{aw}}$ | 2.72 (2.65, 2.79) | 2.13 (1.96, 2.29) | 2.89 (2.84,2.93) | 2.93 (2.87,2.96) | 2.91 (2.82,2.99) | 2.39 (2.27,2.51) |
| $\beta_{\text{traffic}}^{\mathrm{logD}_{aw}}$ | 0.03 (-0.05, 0.10) | -0.14 (-0.31, 0.02) | 0.03 (-0.01,0.07) | 0.03 (-0.01,0.08) | 0.07 (-0.01,0.15) | 0.07 (-0.04,0.18) |
| $\beta_{\text{age}}^{\mathrm{logD}_{aw}}$ | -0.03 (-0.05, 0.10) | 0.06 (-0.20,0.32) | 0.01 (-0.02,0.04) | 0.01 (-0.02,0.03) | 0.000 (-0.05,0.05) | -0.01 (-0.08,0.05) |
| $\beta_{\text{male}}^{\mathrm{logD}_{aw}}$ | 0.09 (0.01, 0.18) | 0.07 (-0.14, 0.28) | 0.02 (-0.03,0.08) | 0.03 (-0.03,0.08) | -0.001 (-0.10,0.10) | 0.01 (-0.12,0.14) |
| $\beta_{\text{asthma}}^{\mathrm{logD}_{aw}}$ | -0.03 (-0.07, 0.02) | 0.05 (-0.05,0.15) | 0.12 (0.06,0.19) | 0.11 (0.04,0.17) | 0.09 (-0.04,0.21) | 0.15 (-0.01,0.31) |

^*^ TS-NLME(*J’_aw_*) is the previously published model using a *J’_aw_* parameterization of TS-NLME.

# **Section 5. Simulation study results using all available estimates (not limited to datasets for which all methods converged).**

**Supplementary Figure 5:** Relative bias (a), coverage (b), and power (c) CI length (d) for $\boldsymbol{\beta}'s$ using all available estimates (not just the subset of estimates from datasets for which all methods converged) for the selected estimation methods in simulation study Scenario 1 ($\beta_{C_{A}}$= $\beta_{{logC}_{aw}}$= $\beta_{{logD}_{aw}}$).


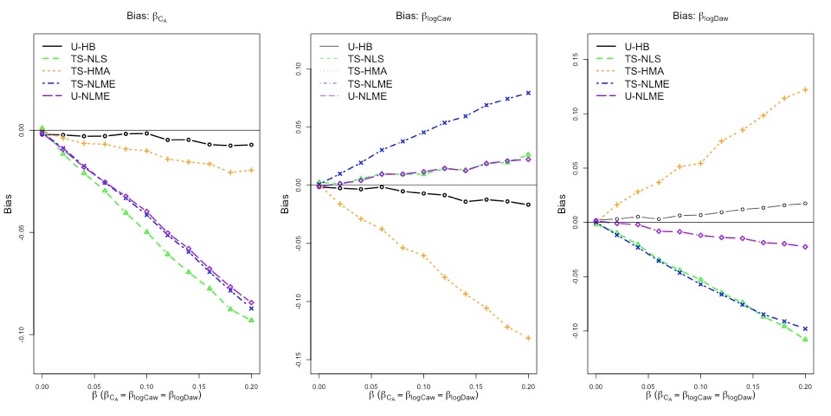

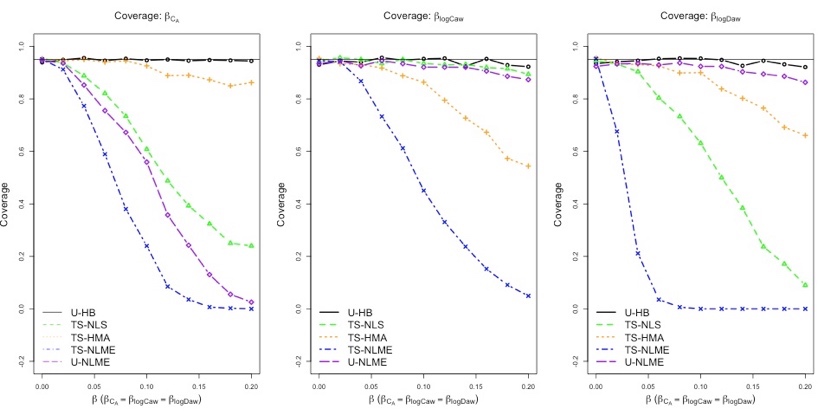

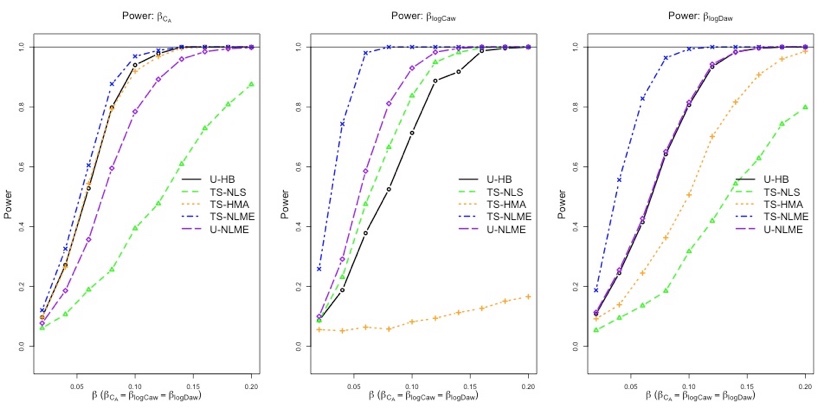

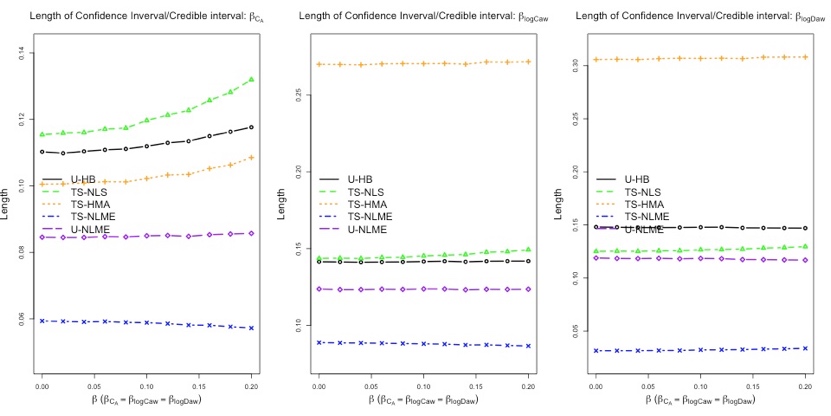


Previously presented results used the subset of simulated datasets on which all methods converged. Since convergence failure rates varied across methods (e.g., ~30% for U-NLME), we present Supplementary Figure 5 including results from simulation Scenario 1 on all available estimates (from all datasets rather than the subset of datasets where all methods converged) to evaluate the impact of this choice on our conclusions. Conclusions for Scenario 1 from Supplementary Figure 5 (all available estimates) are similar to those in Figure 2 (subset of estimates). For other scenarios, the conclusions are also same (data not shown).
